# LRRC4 Orchestrates AP2A1‐Containing Clathrin‐Coated Vesicles to Disrupt Mitochondrial Cristae and Restrict Glioblastoma Progression

**DOI:** 10.1002/advs.76465

**Published:** 2026-07-13

**Authors:** Yang Li, Cheng Huang, Liangqi Jiang, Zhen Li, Qing Liu, Minghua Wu

**Affiliations:** ^1^ Department of Neurosurgery Xiangya Hospital Central South University Changsha Hunan China; ^2^ Clinical Research Center For Skull Base Surgery and Neurooncology in Hunan Province Changsha Hunan China; ^3^ Key Laboratory of Carcinogenesis and Cancer Invasion of Ministry of Education Cancer Research Institute Central South University Changsha Hunan China

**Keywords:** clathrin‐coated vesicles, glioblastoma, LRRC4, mitochondria

## Abstract

Mitochondrial dynamics and metabolic homeostasis are pivotal for the aggressive progression of glioblastoma (GBM); however, how membrane‐bound organelles modulate mitochondrial remodeling remains unclear. In this study, we identified LRRC4 as a Golgi‐anchored tumor suppressor that coordinates a novel vesicle‐to‐mitochondrial axis to inhibit tumor growth. Using single‐sample gene set enrichment analysis (ssGSEA), we established a mitoDynamic score and identified LRRC4 as a key downregulated regulator associated with poor prognosis. Mechanistic analyses combining transmission electron microscopy, Blue Native PAGE, showed that LRRC4 engages AP2A1‐containing clathrin‐coated vesicles in the Golgi apparatus and redirects them to the inner mitochondrial membrane. This targeted vesicular recruitment disrupted the MICOS complex, suppressing Mic60 expression, resulting in cristae collapse, respiratory chain destabilization, and impaired oxidative phosphorylation. Functional assays and xenograft models have demonstrated that structural and metabolic remodeling triggers excessive mitophagy, depletes cellular metabolic fitness, and suppresses GBM proliferation and invasion. Integrating mitoDynamic scoring with mechanistic and rescue experiments established the LRRC4–AP2A1–Mic60 axis as a validated mitochondrial vulnerability. Collectively, these findings revealed a previously unrecognized Golgi mitochondria crosstalk, demonstrated that clathrin‐coated vesicles can act as active modulators of mitochondrial architecture, and suggested that targeting Golgi‐derived clathrin‐coated vesicle‐mediated mitochondrial remodeling is a novel therapeutic strategy for GBM.

## Graphical abstract

LRRC4 suppresses GBM by regulating AP2A1‐containing Golgi‐derived clathrin‐coated vesicles. Low LRRC4 allows cytoplasmic AP2A1 to maintain mitochondrial fission‐fusion, MICOS integrity, and OXPHOS, promoting proliferation and invasion. High LRRC4 redirects AP2A1‐containing Golgi‐derived clathrin‐coated vesicles to mitochondria, disrupts MICOS, enhances fission/mitophagy, and impairs metabolism, limiting GBM growth.

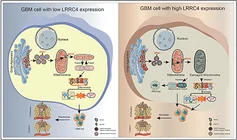

**Abbreviations**
LRRC4: leucine‐rich repeat containing 4
GBM: glioblastoma
N/C Ratio: nuclear to cytoplasmic ratio
MS: mass spectrometry
BP: biological processes
CC: cellular components
MF: molecular functions
IF: immunofluorescence
SDS‐PAGE: sodium dodecyl sulfate polyacrylamide gel electrophoresis
MMP: mitochondrial membrane potential
ROS: reactive oxygen species
LC3: microtubule‐associated protein 1 light chain 3 beta
PINK1: PTEN‐induced putative kinase 1
TOM20: translocase of outer mitochondrial membrane 20
S‐OPA: short OPA1 isoform
OCR: oxygen consumption rate
ECAR: extracellular acidification rate
TCA Cycle: tricarboxylic acid cycle
FMN: flavin mononucleotide
G6P: glucose‐6‐phosphate
F‐1,6P: fructose‐1,6‐bisphosphate
AMP: adenosine monophosphate
GMP: guanosine monophosphate
GTP: guanosine triphosphate
ADP: adenosine diphosphate
BN‐PAGE: blue native polyacrylamide gel electrophoresis
ER: endoplasmic reticulum
AP2A1: adaptor protein complex 2 alpha 1 subunit
AP‐2: adaptor protein 2
OXPHOS: oxidative phosphorylation
MICOS: mitochondrial contact site and cristae organizing system

## 1 Introduction

Glioblastoma (GBM) is the most common malignant tumor of the human central nervous system, accounting for 49% of all primary malignant brain tumor [1]. Maximal tumor resection combined with radiotherapy and temozolomide chemotherapy is the primary treatment option for patients with GBM. However, the prognosis of patients with GBM remains poor, with a median survival of less than 2 years and a 5‐year survival rate of only 5% [2, 3, 4]. Mitochondrial dynamics involve a tightly regulated cycle of morphological remodeling, governed primarily by the opposing actions of dynamin‐related protein‐1 (Drp1)‐mediated fission and Mfn1/2/OPA1‐mediated fusion. This structural equilibrium is frequently compromised [5, 6]. Such dysregulation is now recognized as a fundamental driver of metabolic adaptation and aggressive progression that defines GBM malignancy [7, 8, 9, 10, 11, 12]. Mechanistically, moderate mitochondrial fission facilitates metabolic reprogramming and induces adaptive mitophagy, which maintains mitochondrial quality and collectively exacerbates tumor aggressiveness [7, 8, 9, 10, 11, 12, 13]. Therefore, elucidating the molecular and organellar mechanisms controlling mitochondrial dynamics may provide novel therapeutic opportunities for the treatment of GBM.

Mitochondria are highly dynamic organelles that undergo cycles of fusion and fission, shaping the mitochondrial network, regulating interactions with membrane‐bound organelles, and maintaining mitochondrial function [6, 14]. Drp1 is a key regulator of mitochondrial fission and is recruited to and oligomerized at endoplasmic reticulum (ER)‐mitochondria contact sites, where it mediates constriction to complete membrane scission [15]. Drp1 is highly expressed in glioma stem cells, leading to increased mitochondrial fission, thereby promoting the growth of these cells. Drp1 knockdown induces apoptosis of glioma stem cells and inhibits glioma growth in murine models [9]. In gliomas, overexpression of heat shock protein 90 and calcineurin mediates the dephosphorylation of Drp1 at serine 637, which alters mitochondrial morphology and enhances Acsl4‐mediated lipid peroxidation, thereby promoting ferroptosis [16]. Thus, identifying the regulatory factors or organelles that modulate Drp1 activity could profoundly influence GBM growth and invasion.

Previous studies have established that membrane‐bound organelles such as ER and lysosomes can directly interact with mitochondria through Drp1 to modulate mitochondrial morphology and dynamics [17, 18, 19, 20]. However, whether the Golgi apparatus participates in the regulation of mitochondrial fission and fusion remains unclear. The biological functions of the Golgi apparatus are largely dependent on the vesicles that are active in the cytoplasm. Intra‐Golgi vesicles can be categorized into three types based on their proteinaceous coats and transport vectors: ER‐derived COPII vesicles for anterograde transport, COPI‐coated vesicles for retrograde retrieval from the Golgi back to the ER, and clathrin‐coated vesicles (CCVs) that mediate trafficking from the trans‐Golgi network (TGN) to distal targets [21]. Recent studies revealed a potential link between Golgi‐derived vesicular trafficking and mitochondrial dynamics. Nagashima et al. demonstrated that PI(4)P‐containing vesicles in the trans‐Golgi network can be recruited to mitochondria‐ER contact sites, facilitating Drp1‐mediated mitochondrial fission [22]. The inhibition of PI(4)P production leads to excessive mitochondrial fusion [22]. This discovery identified Golgi‐derived COPI‐coated vesicles as unrecognized modulators of mitochondrial morphology. Golgi‐associated proteins, such as VPS13B, deliver PI(4)P lipids to Mfn2‐positive mitochondrial contact sites, promoting fission and quality control under stress conditions [23]. However, the roles of clathrin‐ and COPII‐coated vesicles in mitochondrial fission and fusion remain unclear.

Leucine‐rich repeat containing 4 (LRRC4) is a tumor suppressor gene in glioma and a member of the leucine‐rich repeat (LRR) superfamily [24, 25]. Classical studies have indicated that cytoplasmic LRRC4 modulates signaling cascades, such as the MEK/ERK pathway, to restrain malignancy [26, 27]. Notably, LRRC4 has been characterized as a membrane‐associated transcription factor that can retrograde through the ER‐Golgi pathway and translocate to the nucleus as a full‐length protein to repress oncogenic target gene expression [28]. However, the extent to which LRRC4 exerts parallel tumor‐suppressive effects at the organelle level independent of its transcriptional activity remains to be explored. In this study, we identified a previously unrecognized function of Golgi‐anchored LRRC4 in orchestrating inter‐organelle communication by controlling clathrin‐coated vesicle transport to the mitochondria. We showed that LRRC4 promotes ubiquitination and redirects AP2A1‐containing Golgi vesicles toward the mitochondrial inner membrane. This unusual vesicle trafficking suppresses Mic60 expression and oligomerization, leading to crista collapse, respiratory chain disassembly, impaired TCA cycle activity, and reduced nucleotide biosynthesis. The resulting structural and metabolic disruptions restrict GBM cell proliferation and invasion. This finding contradicts the traditional view that Golgi vesicles only regulate outer mitochondrial membrane (OMM) fission, and demonstrates that vesicle redirection can function as a tumor‐suppressive metabolic checkpoint.

## 2 Results

### 2.1 Golgi‐Localized LRRC4 Couples Mitochondrial Dynamics to Prognosis of Patients With GBM

To investigate the clinical relevance of mitochondrial dynamics in GBM, we computed a mitochondrial dynamics (mitoDynamic) score in the TCGA‐GBM dataset using single‐sample gene set enrichment analysis (ssGSEA) based on a curated gene set comprising established regulators of mitochondrial fission and fusion [14]. The optimal cutoff for stratifying patients into high‐ and low‐score groups was determined using the surv_cutpoint() function (survminer package, v0.5.1) to maximize survival differences. High scores indicated preserved mitochondrial remodeling, whereas low scores indicated impaired dynamics. Kaplan–Meier analysis revealed that patients with low mitoDynamic scores exhibited significantly shorter overall survival than those with high scores (Figure 1A). Subgroup analysis showed that the mitoDynamic score was lower in mesenchymal and classical GBM subtypes compared with proneural tumors (Figure 1B) and in IDH1‐wild‐type versus mutant tumors (Figure 1C), both of which are associated with aggressive GBM phenotypes [29, 30]. Univariate and multivariate Cox regression analyses further confirmed that the mitoDynamic score was an independent prognostic indicator of GBM (Figure 1D,E). Together, these results establish the mitoDynamic score as a robust quantitative biomarker that links mitochondrial dynamics to prognosis in patient with GBM.

**FIGURE 1:**
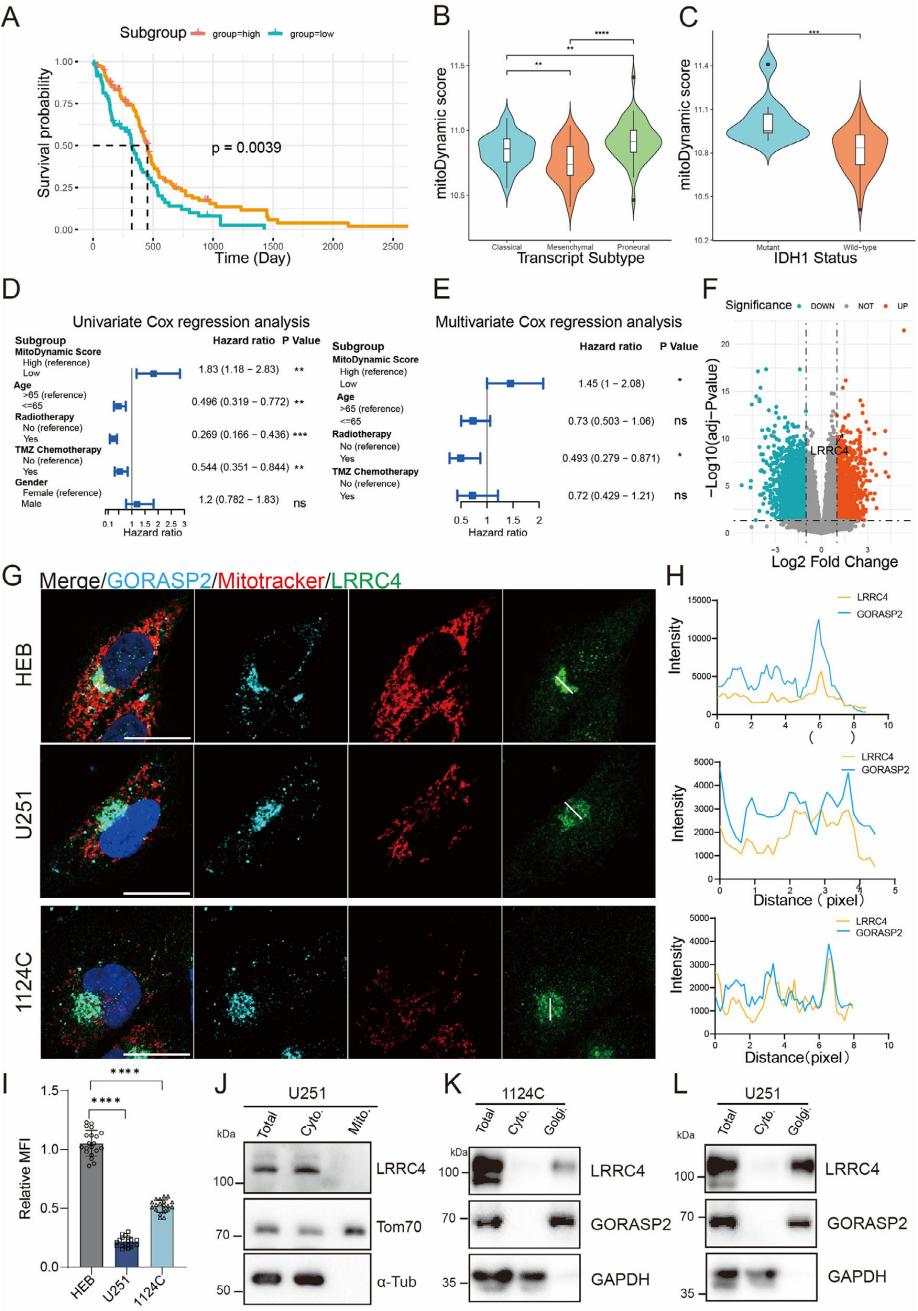
Golgi‐localized LRRC4 correlates with mitochondrial dynamics in GBM. (A) Kaplan‐Meier survival analysis of overall survival in TCGA GBM patients, categorized into high and low mitoDynamic score groups based on the optimal cutoff determined using the survminer R package. (B) Distribution of mitoDynamic scores among GBM transcriptomic subtypes (Classical, Mesenchymal, and Proneural. (C) Comparison of mitoDynamic scores between IDH1‐mutant and IDH1‐wild‐type GBM samples. (D) Forest plot of univariate Cox regression analysis evaluating the association of mitoDynamic score and clinical features (age, radiotherapy, TMZ chemotherapy, and gender) with overall survival. € Multivariate Cox regression analysis incorporating mitoDynamic score and clinical covariates. (F) Volcano plot showing differentially expressed genes between high and low mitoDynamic score groups, with upregulated (orange) and downregulated (green) genes highlighted. LRRC4 is indicated. (G) LRRC4 immunofluorescence (green) shows colocalization with the Golgi apparatus marker GORASP2 (cyan) in HEB, U251, and 1124C cells. Scale = 10 µM. (H) Colocalization of LRRC4 and GORASP2 was analyzed using Image J. (I) The intensity of LRRC4 immunofluorescence (green) was decreased in U251 and 1124C cells compared to HEB. N=30, **** *p* < 0.0001. (J) Mitochondria were extracted from 1124C and U251 cells, and SDS‐PAGE was used to examine the localization of LRRC4. α‐Tubulin (α‐Tub) was used as a cytoplasmic marker, and Tom70 was used as a mitochondrial marker. (K,L) Golgi apparatus were extracted from 1124C and U251 cells, and SDS‐PAGE was used to examine the localization of LRRC4. α‐Tubulin (α‐Tub) was used as a cytoplasmic marker, and GORASP2 was used as a Golgi apparatus marker.

Differential expression analysis between high‐ and low‐mitoDynamic score tumors (Table S1) identified LRRC4 as a top‐ranked gene with significantly higher expression in high‐score tumors associated with better survival (Figure 1F). Although generally downregulated in GBM relative to the normal brain, LRRC4 upregulation in high mitoDynamic tumors suggests a positive link between preserved mitochondrial dynamics and favorable prognosis. Previous studies indicate that LRRC4 exerts tumor‐suppressive effects in GBM by regulating the mitochondrial structure, specifically by transforming mitochondria from spindle to oval shapes to inhibit cell proliferation and invasion [24, 31]. To confirm organelle specificity, we evaluated the structural and functional integrity of ER and lysosomes. LRRC4 overexpression preserved ER and lysosomal integrity without triggering an unfolded protein response, as confirmed by normal organelle architecture and stable expression of stress markers (BIP, CHOP, and XBP1) (Figure S1A–E). To investigate its localization, IF was performed, and we demonstrated that LRRC4 co‐localizes with the Golgi marker GORASP2 in HEB, U251, and 1124C cells rather than in the mitochondria. (Figure 1G,H). Quantitative analysis revealed that LRRC4 expression was markedly reduced in the Golgi apparatus of GBM cells compared to HEB cells (Figure 1I). Subcellular fractionation followed by immunoblotting confirmed that LRRC4 was mainly enriched in the Golgi apparatus rather than in the mitochondria (Figure 1J–L). Bioinformatics analysis of CGGA cohorts and previous studies reveals that LRRC4 reduced expression correlates with poor overall survival of patients with GBM (Figure S1F) [31, 32].

Taken together, these findings indicate that LRRC4 is primarily localized to the Golgi apparatus in glioblastoma cells, and its reduced expression may correlate with poor prognosis and aberrant mitochondrial dynamics of patients with GBM.

### 2.2 LRRC4 Drives Excessive Mitochondrial Fission and Mitophagy in GBM

To investigate the impact of LRRC4 on mitochondrial structure and function, we generated U251 and 1124C GBM cell lines stably expressing LRRC4‐FLAG‐His. IF analysis revealed that LRRC4 localized predominantly to the cytoplasm and Golgi apparatus, and its overexpression markedly increased the proportion of fragmented mitochondria compared to control cells (Figure 2A–B). Quantitative morphometric analysis confirmed that LRRC4 overexpression significantly decreased the mitochondrial length, area, and perimeter, resulting in smaller and more fragmented organelles (Figure 2C). Consistently, immunoblotting revealed the upregulation of the fission regulators Drp1 and OMA1, along with decreased levels of the fusion proteins MFN1 and long OPA1, indicating that LRRC4 enhanced mitochondrial fission (Figure 2D–E).

**FIGURE 2:**
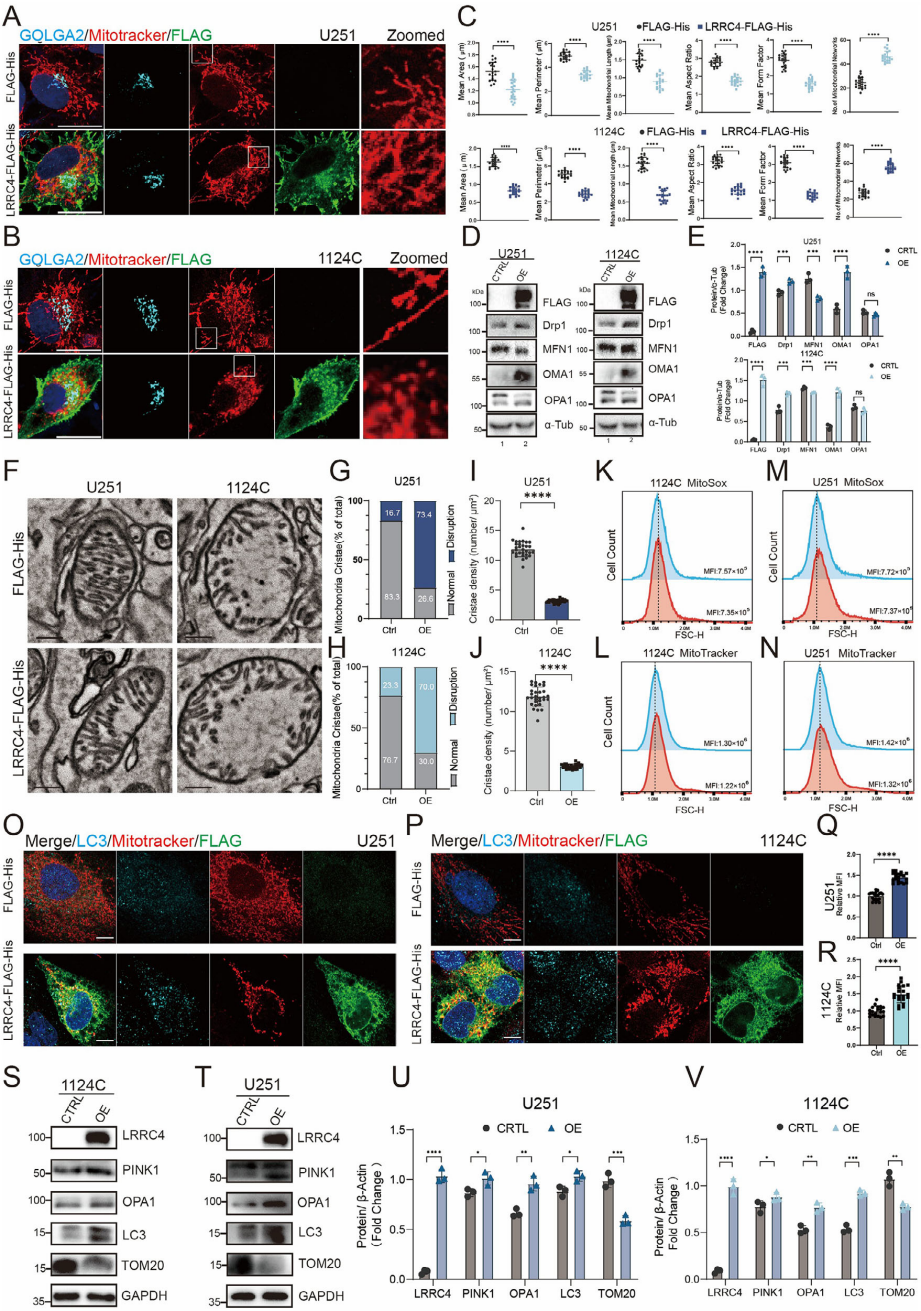
Overexpression of LRRC4 in GBM cells induces mitochondrial fission and cristae rupture. (A,B) 1124C and U251 cells were stably transfected with LRRC4‐FLAG‐His and FLAG‐His. LRRC4‐FLAG immunofluorescence (green) showed partial colocalization with the Golgi marker GOLGA2 (cyan). Expression of LRRC4‐FLAG resulted in an increase in fragmented mitochondrial fission compared with FLAG‐His, indicating that LRRC4 overexpression can induce mitochondrial fission. Scale bar= 10 µM. (C) Quantification of mitochondrial morphology parameters using Image J, including length, area, perimeter, aspect ratio, and form factor, in U251 and 1124C cells after transfected with LRRC4‐FLAG‐His and FLAG‐His (n=20). (D) 1124C and U251 cells were stably transfected with LRRC4‐FLAG‐His (OE) and FLAG‐His (CTRL). SDS‐PAGE of these cell lysates showed the downregulation and upregulation of mitochondrial fission and fusion mediators. (E) Quantitative analysis of the relative protein levels of mitochondrial fission and fusion mediators presented in (D). (F) Transmission electron microscopy was used to observe changes in mitochondrial morphology in U251 and 1124C cells after the overexpression of LRRC4. The mitochondria in LRRC4‐overexpressing GBM cells changed from their normal elongated rod‐like shape to a more rounded form. Additionally, there was a reduction in mitochondrial cristae, and the connections between the cristae and the inner membrane were disrupted. n=30, Scale bar=200 nm. (G,H) Quantitative analysis of mitochondrial cristae percentage (Normal vs. Disruption) in U251 (G) and 1124C (H) cells. I‐J Quantitative analysis of cristae density (number/µm^2^) in U251 (I) and 1124C (J) cells. (K—N) 1124C and U251 cells were stably transfected with LRRC4‐FLAG‐His and FLAG‐His, then stained with 100 nM Mitotracker and 50 µM MitoSox for 30 min. The changes in fluorescence intensity in LRRC4‐overexpressing GBM cells were then analyzed using flow cytometry. (O,P) 1124C and U251 cells were stably transfected with LRRC4‐FLAG‐His (OE) and FLAG‐His (CTRL), the fluorescence intensity of LC3B(cyan) increases with the upregulation of LRRC4(red) expression. (Q,R) Quantitative analysis of LC3B(cyan) fluorescence intensity in the mitochondrial region is presented in (O) and (P). n=20, **** p < 0.0001. (S,T) SDS‐PAGE analysis revealed changes in the expression levels of mitophagy‐related proteins in 1124C and U251 cells between the LRRC4 overexpression group and the control group. (U,V) Quantitative analysis of the relative expression levels of mitophagy‐related proteins (including TOM20) presented in (S) and (T). n=3, *p < 0.05, **p < 0.01, ***p < 0.001, **** *p* < 0.0001.

Transmission electron microscopy (TEM) revealed extensive ultrastructural remodeling in LRRC4‐overexpressing cells. Compared with the elongated, rod‐like mitochondria in control cells, LRRC4‐overexpressing mitochondria were round, swollen, and displayed disrupted cristae with reduced crista‐inner membrane junctions (Figure 2F–J and Figure S2A,B). Given that intact cristae are essential for oxidative phosphorylation (OXPHOS) efficiency, we assessed mitochondrial function [33]. Flow cytometry analysis demonstrated that LRRC4 overexpression reduced the mitochondrial membrane potential and reactive oxygen species (ROS) production, reflecting impaired mitochondrial bioenergetics (Figure 2K–N).

As mitochondrial fragmentation and loss of function are frequently coupled with mitophagy [34], we examined whether LRRC4 activates this process. IF staining showed a striking increase in LC3B puncta co‐localizing with mitochondria in LRRC4‐overexpressing cells (Figure 2O–R), suggesting enhanced mitophagy. These findings were corroborated by immunoblotting, which revealed elevated levels of the mitophagy‐associated proteins PINK1 and LC3‐IIB, accumulation of short OPA1, and a marked reduction in Tom20 (Figure 2S–V). Collectively, these findings demonstrate that LRRC4 overexpression promotes excessive mitochondrial fission, disrupts cristae organization, and activates mitophagy, thereby remodeling the mitochondrial structure and function in GBM cells.

### 2.3 LRRC4 Disrupts Respiratory Chain Supercomplex Assembly and Mitochondrial Energy Metabolism

Mitochondrial cristae are sites of oxidative phosphorylation, and ATP production depends on the integrity of respiratory chain complexes in mitochondrial cristae [5, 35]. Given that LRRC4 overexpression disrupts the cristae, we examined its impact on mitochondrial bioenergetics in GBM cells. GSEA of transcriptomic data from LRRC4‐overexpressing U251 cells revealed a significant downregulation of OXPHOS‐related pathways, including the electron transport chain, mitochondrial respiratory chain complex assembly, cytochrome c oxidase assembly, respiratory electron transport, proton motive force‐driven ATP synthesis, and overall oxidative phosphorylation (Figure 3A–F, Table S2). Consistent with these findings, intracellular ATP measurements showed marked reductions in both U251 and 1124C cells (Figure 3G,H), whereas enzymatic activity assays revealed decreased complex I and V activities, confirming impaired oxidative phosphorylation (Figure 3I–L). To further assess mitochondrial respiration, we performed a seahorse extracellular flux analysis. Oxygen consumption rate measurements showed that LRRC4 overexpression led to pronounced decreases in basal respiration, ATP‐linked respiration, and maximal respiration in both cell lines (Figure 3M,N). Quantitative analysis confirmed that spare respiratory capacity and non‐mitochondrial oxygen consumption were also significantly impaired, whereas proton leakage remained largely unchanged in 1124C cells (Figure 3Q,R). Parallel measurements of the extracellular acidification rate revealed that glycolytic activity was similarly reduced in LRRC4‐overexpressing U251 and 1124C cells (Figure 3O,P).

**FIGURE 3:**
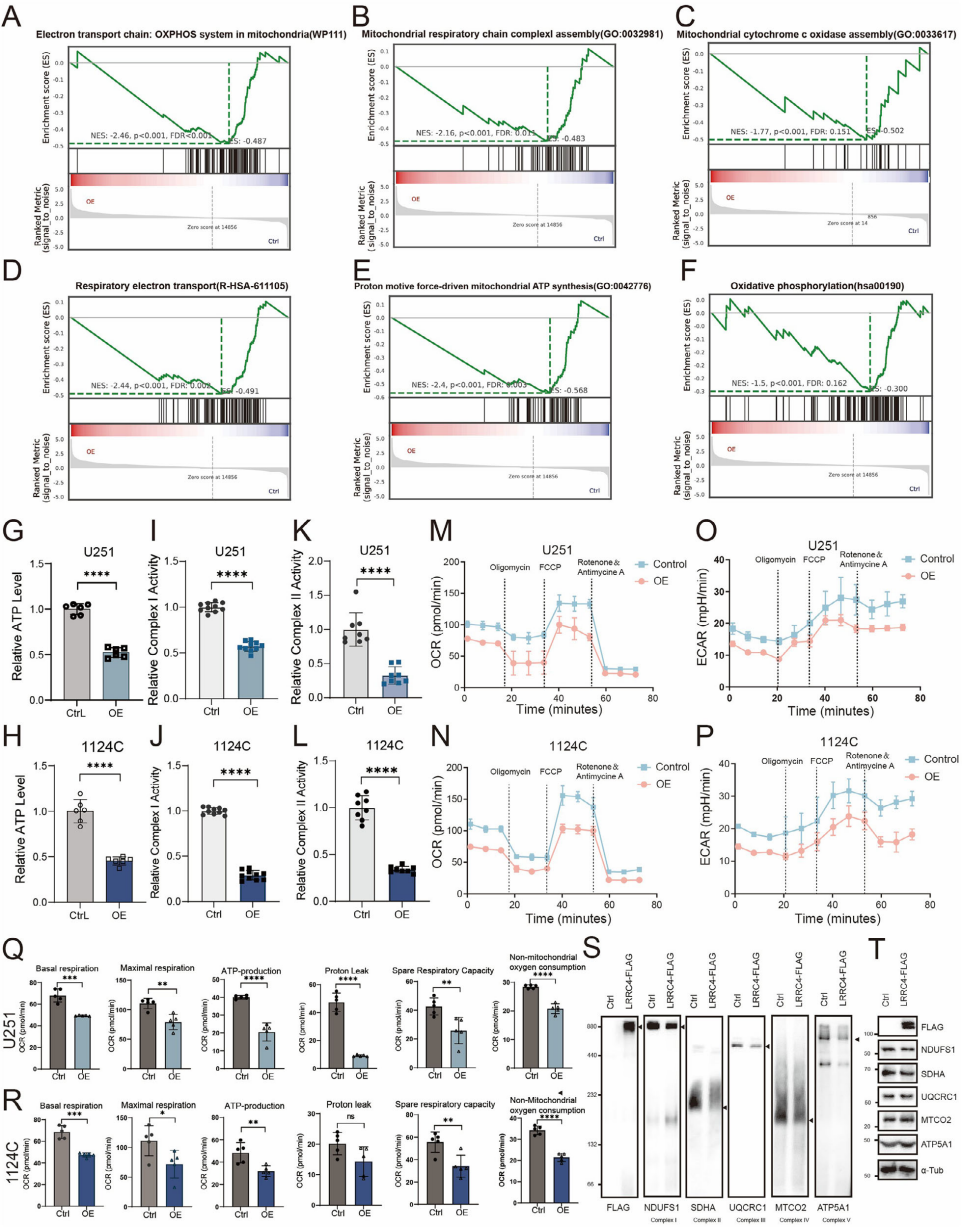
Overexpression of LRRC4 impairs mitochondrial oxidative phosphorylation and reduces ATP production in GBM cells. (A—F) Gene set enrichment analysis (GSEA) of differentially expressed genes from U251 cells overexpressing LRRC4 showed significant downregulation of mitochondrial oxidative phosphorylation‐related pathways, including the electron transport chain (A), mitochondrial respiratory chain complex assembly (B), cytochrome c oxidase assembly (C), respiratory electron transport (D), proton motive force‐driven ATP synthesis (E), and oxidative phosphorylation (F). (G—L) Biochemical assays confirmed that LRRC4 overexpression reduced intracellular ATP levels (G, H), complex I activity (I, J), and complex V activity (K, L) in U251 and 1124C cells compared with controls. (M—P) Oxygen consumption rate (OCR) profiles of U251 and 1124C cells were measured using a Seahorse XF Analyzer. Sequential addition of oligomycin, FCCP, and rotenone/antimycin A was used to assess mitochondrial respiration, including basal respiration, ATP production, maximal respiration, and spare respiratory capacity(M‐N). Extracellular acidification rate (ECAR) was measured in U251 (O) and 1124C (P) cells to evaluate glycolytic activity. (Q—R) Quantitative comparison of mitochondrial respiration parameters, including basal respiration, maximal respiration, ATP production, proton leak, spare respiratory capacity, and non‐mitochondrial oxygen consumption, in U251 (Q) and 1124C (R) and cells between the control group (Ctrl) and LRRC4 overexpression (OE) group. n = 5, *p < 0.05, **p < 0.01, ***p < 0.001, ns: not significant. (S,T) BN‐PAGE (S) and SDS‐PAGE (T) were performed to assess the expression and assembly of mitochondrial respiratory chain complexes following LRRC4 overexpression in 1124C and U251 cells. Chemiluminescence was used for signal detection. Black arrows indicate the positions of the major native oxidative phosphorylation (OXPHOS) complexes, including NADH:ubiquinone oxidoreductase (complex I, CI; ∼1000 kDa), cytochrome bc1 complex dimer (complex III_2_, CIII_2_; ∼500 kDa), cytochrome c oxidase (complex IV, CIV; ∼220 kDa), and ATP synthase (complex V, CV; ∼660 kDa).

To determine whether these defects arose from structural alterations in respiratory chain complexes, we performed blue native PAGE (BN‐PAGE) combined with SDS‐PAGE. While the expression of individual respiratory chain subunits remained largely unaltered, the assembly of complexes I, III, IV, and V was markedly disrupted in LRRC4‐overexpressing cells, indicating the instability of the supercomplex organization (Figure 3S–T and Figure S3A–B). Collectively, these data demonstrated that LRRC4 overexpression compromised the structural integrity of mitochondrial respiratory chain supercomplexes, leading to impaired OXPHOS, reduced ATP production, and broad reprogramming of cellular energy metabolism in GBM cells.

### 2.4 LRRC4 Suppresses Glycolysis, TCA Flux and Nucleotide Biosynthesis

Given that LRRC4 disrupts the assembly of respiratory chain complexes and oxidative phosphorylation, we investigated its effects on mitochondrial metabolism. Transcriptomic analysis (RNA‐seq) of LRRC4‐overexpressing U251 cells revealed a significant downregulation of genes associated with the TCA cycle, respiratory electron transport, and aerobic respiration pathways (Figure 4A–D). To functionally validate these findings, we performed targeted metabolomic analysis in LRRC4‐overexpressing U251 and 1124C cells. The resulting heatmap revealed extensive metabolic alterations, particularly in glycolysis, the TCA cycle, and nucleotide biosynthesis pathways (Figure 4E). LRRC4 overexpression markedly decreased key glycolytic intermediates, including glucose‐6‐phosphate; fructose‐6‐phosphate; fructose‐1,6‐bisphosphate; and lactate levels, indicating a strong suppression of glycolytic flux (Figure 4F). Meanwhile, TCA intermediates such as citrate, malate, and succinate were significantly reduced, accompanied by decreased NAD^+^/NADH ratios and lower flavin mononucleotide levels, reflecting impaired mitochondrial redox capacity and oxidative phosphorylation (Figure 4F). Moreover, LRRC4 overexpression led to a substantial decline in GMP, AMP, and related nucleotides, indicating the disruption of purine metabolism and nucleotide synthesis (Figure 4G). Collectively, these results demonstrate that LRRC4 overexpression induces profound metabolic reprogramming in GBM cells, characterized by the transcriptional and metabolic suppression of glycolysis, the TCA cycle, and nucleotide synthesis. This effect may be driven by the disruption of MICOS complex integrity and mitochondrial cristae architecture, thereby constraining both bioenergetic output and anabolic capacity.

**FIGURE 4:**
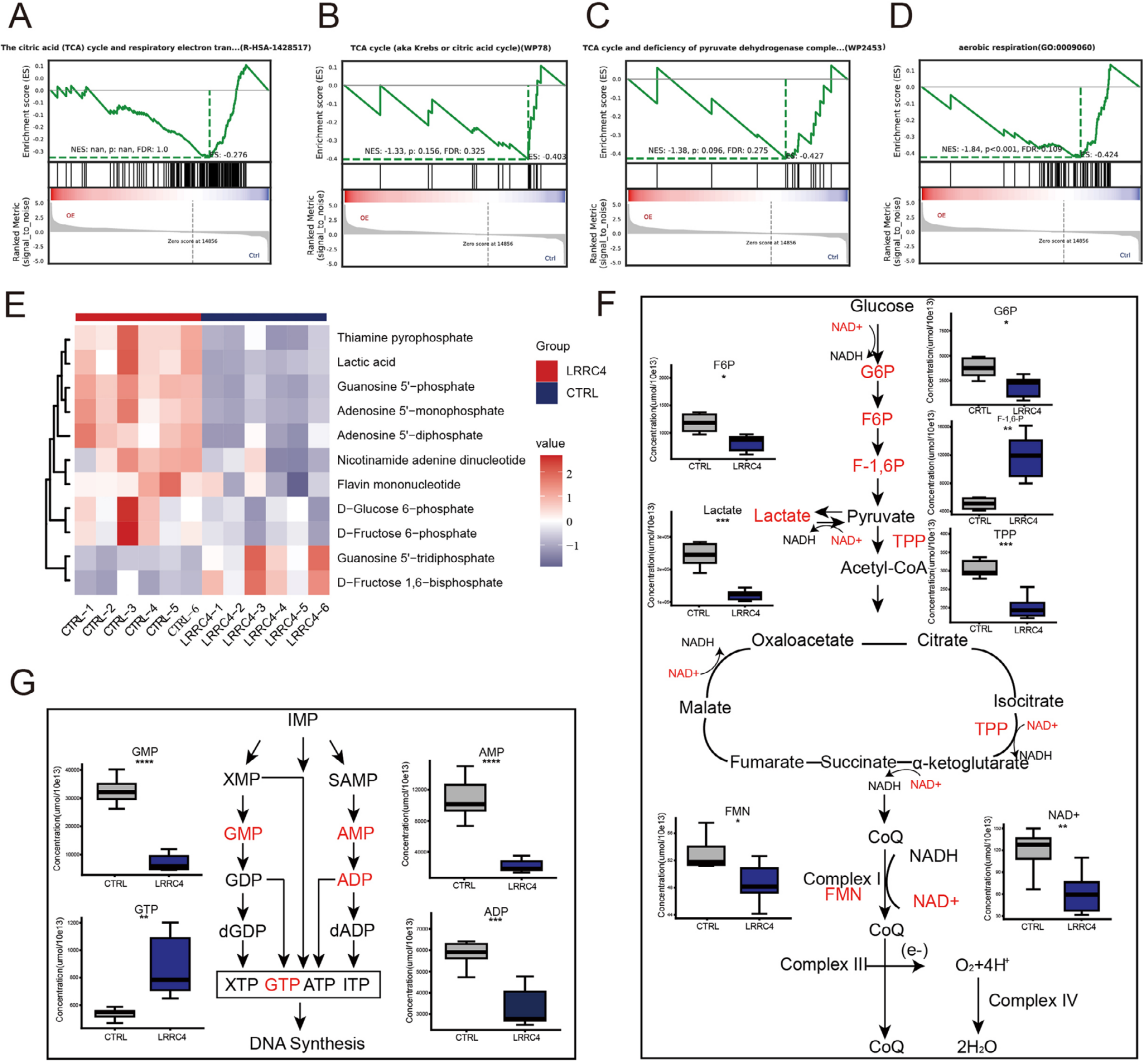
LRRC4 overexpression remodels TCA cycle activity and nucleotide metabolism in GBM cells. Gene set enrichment analysis (GSEA) of differentially expressed genes from U251 cells overexpressing LRRC4 revealed significant downregulation of pathways associated with the TCA cycle and respiratory electron transport (A), TCA cycle/citric acid cycle (B), pyruvate dehydrogenase complex deficiency (C), and aerobic respiration (D). E Heatmap of targeted metabolomics profiling showing altered metabolites involved in glycolysis, TCA cycle, and nucleotide metabolism between LRRC4‐overexpressing (OE) and control (CTRL) cells (n=6). F Schematic representation of glycolysis and the TCA cycle with quantified metabolite levels (box plots) indicating that LRRC4 overexpression reduced intermediates such as glucose‐6‐phosphate (G6P), fructose‐6‐phosphate (F6P), fructose‐1,6‐bisphosphate (F‐1,6P), thiamine pyrophosphate (TPP), and NAD+/NADH balance, while increasing lactate production (n=4). G Pathway analysis of purine metabolism demonstrated that LRRC4 overexpression disrupted nucleotide synthesis, with decreased GMP, AMP, ADP, and GTP levels in U251 cells compared with controls (n=4). Data are presented as mean ± SD, * *p* < 0.05, ** *p* < 0.01, *** *p* < 0.001, **** *p* < 0.0001.

### 2.5 LRRC4 Interacts With AP2A1 to Control Mitochondrial Fission

To elucidate the downstream molecular effectors through which Golgi‐localized LRRC4 modulates mitochondrial dynamics, we first performed unbiased LC‐MS/MS proteomic analysis of U251 cells stably overexpressing LRRC4. Differentially expressed proteins (fold change >1.5, *p* < 0.05) were identified. To narrow our search for Golgi‐related mechanisms, we compared these DEPs with a curated list of Golgi apparatus‐associated genes (obtained from the gene set enrichment analysis database). Bioinformatic filtering yielded eight overlapping candidates. Among these, AP2A1, which encodes the alpha‐adaptin subunit of the clathrin‐associated adaptor protein 2 (AP‐2) complex [36, 37], emerged as a top candidate for further investigation because of its significant upregulation and established role in intracellular trafficking (Figure 5A, Table S3). Western blotting demonstrated that LRRC4 directly binds with AP2A1 and concomitantly reduces its endogenous protein levels in both U251 and 1124C cells (Figure 5B,C). LRRC4 overexpression promoted AP2A1 polyubiquitination, targeting it for proteasome‐mediated degradation, as evidenced by the restoration of AP2A1 levels after MG132 treatment (Figure 5D,E). These results established that LRRC4 governs AP2A1 abundance via a ubiquitin‐dependent pathway.

**FIGURE 5:**
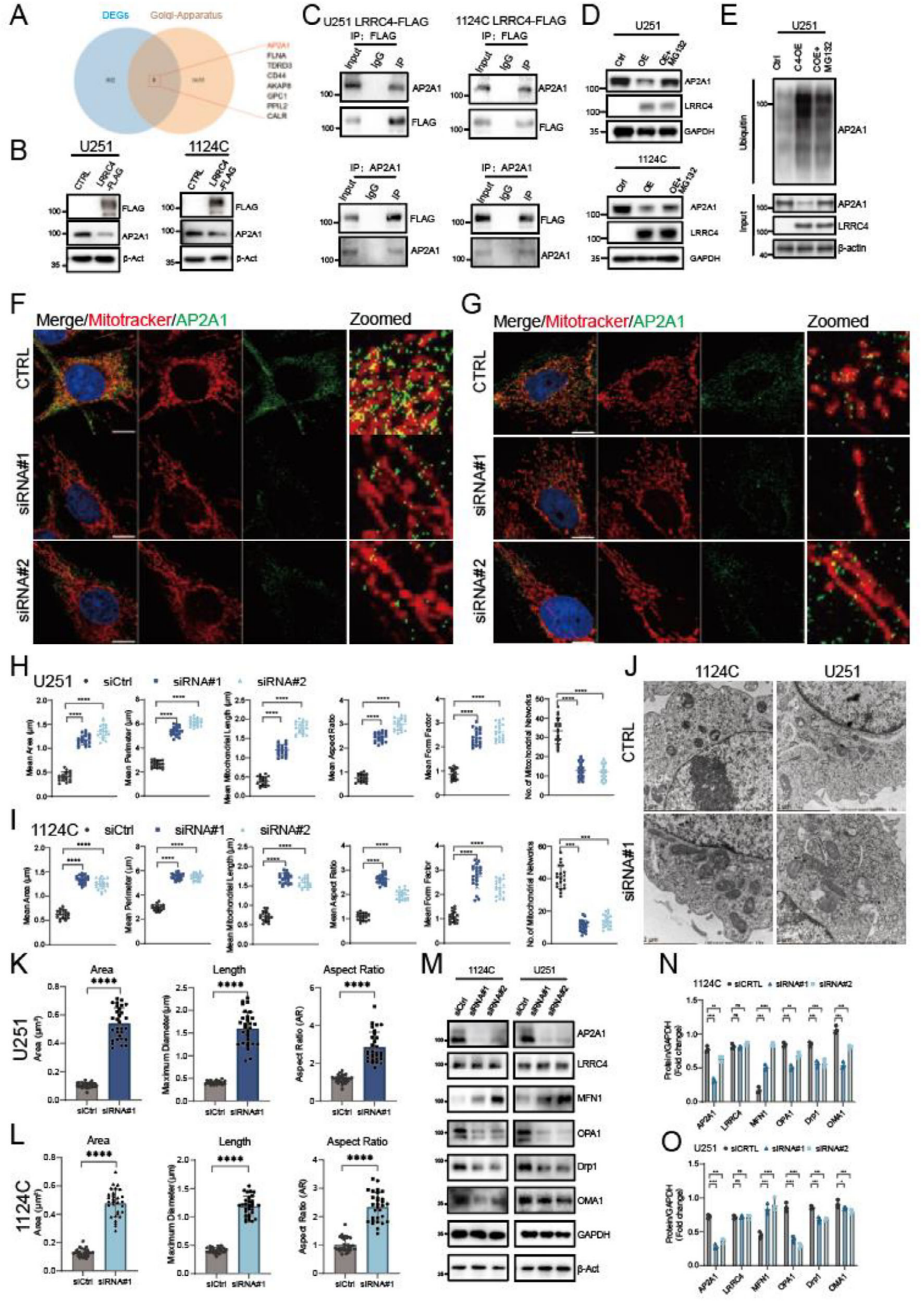
LRRC4 interacts with AP2A1 and promotes its ubiquitination‐mediated degradation to regulate mitochondrial fission. (A) Venn diagram of differentially expressed genes (DEGs) from U251 cells overexpressing LRRC4 compared with the control group, intersected with Golgi‐associated genes (MSigDB, https://www.gsea‐msigdb.org/gsea/msigdb), identified eight overlapping candidates, including AP2A1, a core subunit of the adaptor protein complex 2 (AP2). (B) Western blot analysis confirmed that LRRC4‐FLAG overexpression reduces endogenous AP2A1 protein levels in both U251 and 1124C cells. C Western blot analysis confirmed that LRRC4‐FLAG overexpression reduces endogenous AP2A1 protein levels in both U251 and 1124C cells. (D,E) LRRC4 overexpression was found to promote the polyubiquitination of AP2A1 (E), leading to its proteasome‐dependent degradation, as evidenced by the rescue of AP2A1 levels following MG132 treatment (D). (F,G) Immunofluorescence staining of AP2A1 (green) and Mitotracker (red) in U251 (E) and 1124C (F) cells demonstrated that AP2A1 knockdown (siRNA#1/siRNA#2) promoted mitochondrial elongation and fusion compared with siCtrl. Scale bar = 10 µm. (H,I) Quantitative analysis of mitochondrial morphology parameters in (E,F) (length, area, perimeter, aspect ratio, form factor) revealed significant elongation and increased fusion after AP2A1 knockdown in U251 (G) and 1124C (H) cells (n=20). (J—L) Transmission electron microscopy and corresponding statistical analysis verified that silencing AP2A1 leads to an elongated mitochondrial ultrastructure with increased area, maximum diameter, and aspect ratio (Scale bar = 2 µm). (M—O) Immunoblotting and relative protein quantification demonstrated that AP2A1 knockdown modulates mitochondrial dynamics by downregulating fission mediators (Drp1, OMA1) and upregulating fusion mediators (MFN1). Data are presented as mean ± SD from at least three independent biological replicates (* *p* < 0.05, *p* < 0.01, *** *p* < 0.001, **** *p* < 0.0001).

Mitochondrial fission requires the involvement of the ER and lysosomes [38], and recent evidence indicates that Golgi‐derived vesicles contribute to the late stages of mitochondrial division [39]. Based on these insights, we examined the functional consequences of AP2A1 depletion by transfecting U251 and 1124C cells with two independent AP2A1‐targeting siRNAs followed by mitochondrial staining. IF revealed that AP2A1 knockdown promoted mitochondrial elongation and enhanced network fusion compared with siCtrl cells (Figure 5F.G). Quantitative analysis revealed significant increases in mitochondrial length, area, and aspect ratio, confirming the reduced fission activity (Figure 5H,I). Transmission electron microscopy further validated that AP2A1 silencing led to elongated mitochondria with an increased area, maximum diameter, and aspect ratio (Figure 5J–L). Western blotting demonstrated that AP2A1 knockdown decreased the fission mediators Drp1 and OMA1, while upregulating the fusion mediator MFN1 without affecting LRRC4 levels (Figure 5M–O). Taken together, these data indicate that LRRC4 interacts with AP2A1 and promotes its ubiquitination‐mediated degradation, thereby regulating mitochondrial fission and network morphology in GBM cells.

### 2.6 LRRC4 Recruits AP2A1‐Clathrin Vesicles to Mitochondria and Disrupts MICOS

Clathrin‐coated vesicles are typically involved in mediating trafficking between the trans‐Golgi network, the plasma membrane, and endosomes. To assess whether AP2A1‐containing CCVs participate in Golgi‐mitochondria communication, we examined their localization upon LRRC4 overexpression in U251 and 1124C cells. IF demonstrated that LRRC4 overexpression significantly enhanced the colocalization of AP2A1 and clathrin‐positive vesicles with mitochondria in both GBM cells, suggesting that these vesicles were actively recruited to the mitochondria (Figure 6A–D). Quantitative analysis of the mean fluorescence intensity within the mitochondrial region showed an elevated FLAG signal for LRRC4 and increased AP2A1 intensity, indicating active recruitment and accumulation in the mitochondria (Figure 6E,F).

**FIGURE 6:**
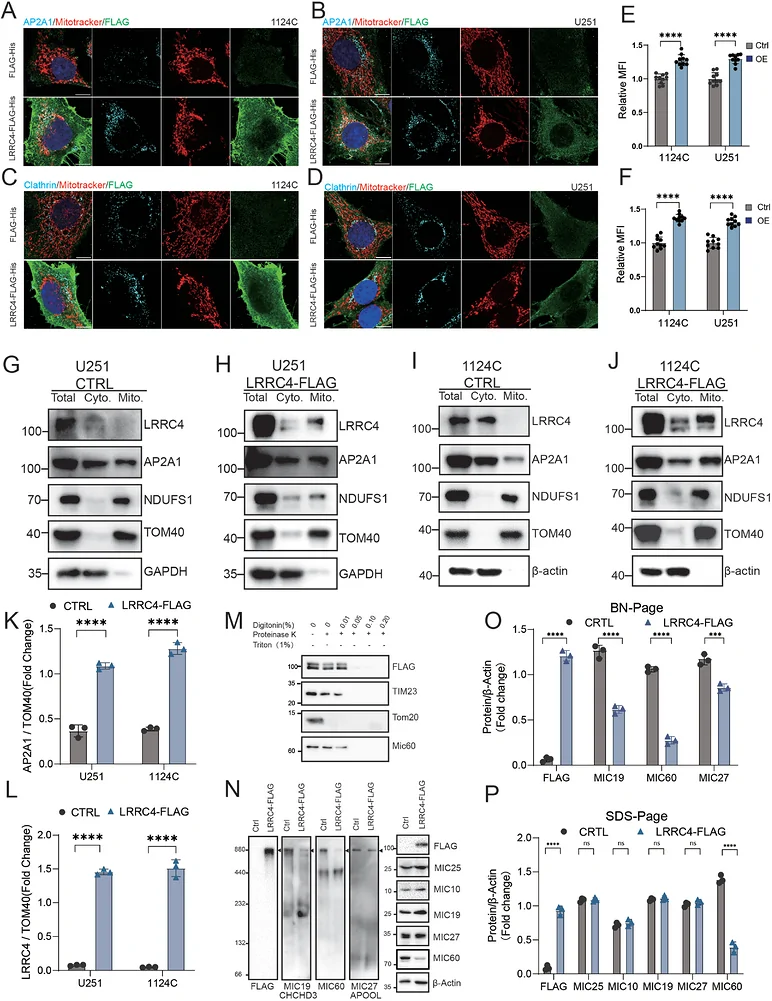
LRRC4 recruits AP2A1‐clathrin vesicles to mitochondria and disrupts MICOS in GBM. (A,B) Immunofluorescence staining of AP2A1 (cyan), Mitotracker (red), and LRRC4‐FLAG (green) in 1124C (A) and U251 (B) cells. LRRC4 overexpression increased the colocalization of AP2A1‐positive vesicles with mitochondria. (C,D) Immunofluorescence staining of clathrin (cyan), Mitotracker (red), and LRRC4‐FLAG (green) in 1124C (C) and U251 (D) cells. LRRC4 overexpression enhanced the recruitment of clathrin‐coated vesicles to mitochondria. Scale bar = 10 µm. (E,F) Quantification of mitochondrial‐localized FLAG (E) and AP2A1 (F) Mean Fluorescence Intensity (MFI) confirms significant accumulation of these proteins at the mitochondria. (G—J) Subcellular fractionation and immunoblot analysis demonstrating the distribution of LRRC4 and AP2A1 in U251 and 1124C cells. Densitometric quantification of target protein bands in the mitochondrial (Mito.) fraction was performed using ImageJ software (Fiji). Integrated density values for each band were background‐subtracted and normalized to the intensity of mitochondrial markers (TOM40 or NDUFS1) to control for variations in protein loading. The mitochondrial enrichment ratio was calculated as the normalized mitochondrial signal relative to the total lysate signal. Data are presented as the mean ± SD from at least three independent biological replicates. Statistical significance between groups was determined using an unpaired Student's *t*‐test. **** *p* < 0.0001. (K,L) Densitometric quantification of AP2A1 (K) and LRRC4 (L) normalized to the mitochondrial marker TOM40 validates their enrichment in the mitochondrial fraction upon LRRC4 overexpression. (M) Proteinase K (PK) protection assay was performed using purified mitochondria from LRRC4‐overexpressing U251 cells. While the outer membrane marker Tom20 was susceptible to PK digestion at low digitonin concentrations, the degradation pattern of LRRC4‐FLAG mirrored those of the inner membrane markers TIM23 and Mic60. These results confirm the localization of LRRC4 to the mitochondrial inner membrane. (N) Blue native PAGE (BN‐PAGE) and SDS‐PAGE analysis showed that LRRC4 overexpression disrupted the integrity of the MICOS complex and altered the expression of its subunits (MIC19, CHCHD3, MIC60, MIC27, APOOL), indicating impaired cristae organization. (O,P) Quantitative analysis of the BN‐PAGE (O) and SDS‐PAGE (P) results confirms the reduced assembly of MICOS subcomplexes and significantly decreased expression of the MIC60 subunit. Scale bar = 10 µm. Data are presented as mean ± SD; **** *p* < 0.0001, ns: not significant.

Subcellular fractionation followed by immunoblotting revealed a marked increase in the proportion of AP2A1 and LRRC4 localized to the mitochondrial fraction upon LRRC4 overexpression (Figure 6G–J). Densitometric quantification normalized to the mitochondrial marker TOM40 validated the significant enrichment of these proteins within the mitochondrial fraction of both U251 and 1124C cells (Figure 6K,L). To precisely define the submitochondrial localization of LRRC4, we performed a proteinase K (PK) protection assay on purified mitochondria from LRRC4 overexpressed U251. Although the outer membrane marker Tom20 was susceptible to PK digestion at low digitonin concentrations, LRRC4 remained protected, mirroring the degradation patterns of the inner membrane markers TIM23 and Mic60 (Figure 6M). These data confirmed that the overexpressed LRRC4 was mainly localized to the mitochondrial inner membrane.

We next evaluated the downstream effects of this vesicular trafficking on mitochondrial organization, BN‐PAGE and SDS‐PAGE analyses revealed that LRRC4 overexpression in U251 disrupted MICOS complex integrity, as evidenced by reduced assembly of both MIC10‐MIC26‐MIC27 and MIC60‐MIC19‐MIC25 subcomplexes, with the latter defect linked to decreased MIC60 expression (Figure 6N–P). Given the essential role of the MICOS complex in maintaining cristae junctions and the inner membrane structure [40, 41], these findings suggest that LRRC4‐driven recruitment of AP2A1‐containing vesicles to mitochondria directly impairs cristae organization.

### 2.7 Mic60 Restoration Rescues LRRC4‐Induced Mitochondrial Defects and Growth Suppression

To determine whether MICOS disruption functionally contributes to LRRC4‐mediated mitochondrial remodeling, we re‐expressed Mic60 in LRRC4‐overexpressing GBM cells. BN‐PAGE revealed that LRRC4 overexpression markedly impaired the assembly of MICOS‐related complexes, including Mic60‐, Mic19‐, Mic27‐, Mic25‐, and Mic10‐containing complexes, whereas Mic60 restoration partially rescued MICOS assembly (Figure 7A,C). Consistently, SDS‐PAGE confirmed that LRRC4 reduced Mic60 and associated MICOS protein levels, which were partially restored by Mic60 re‐expression (Figure 7B,D).

**FIGURE 7:**
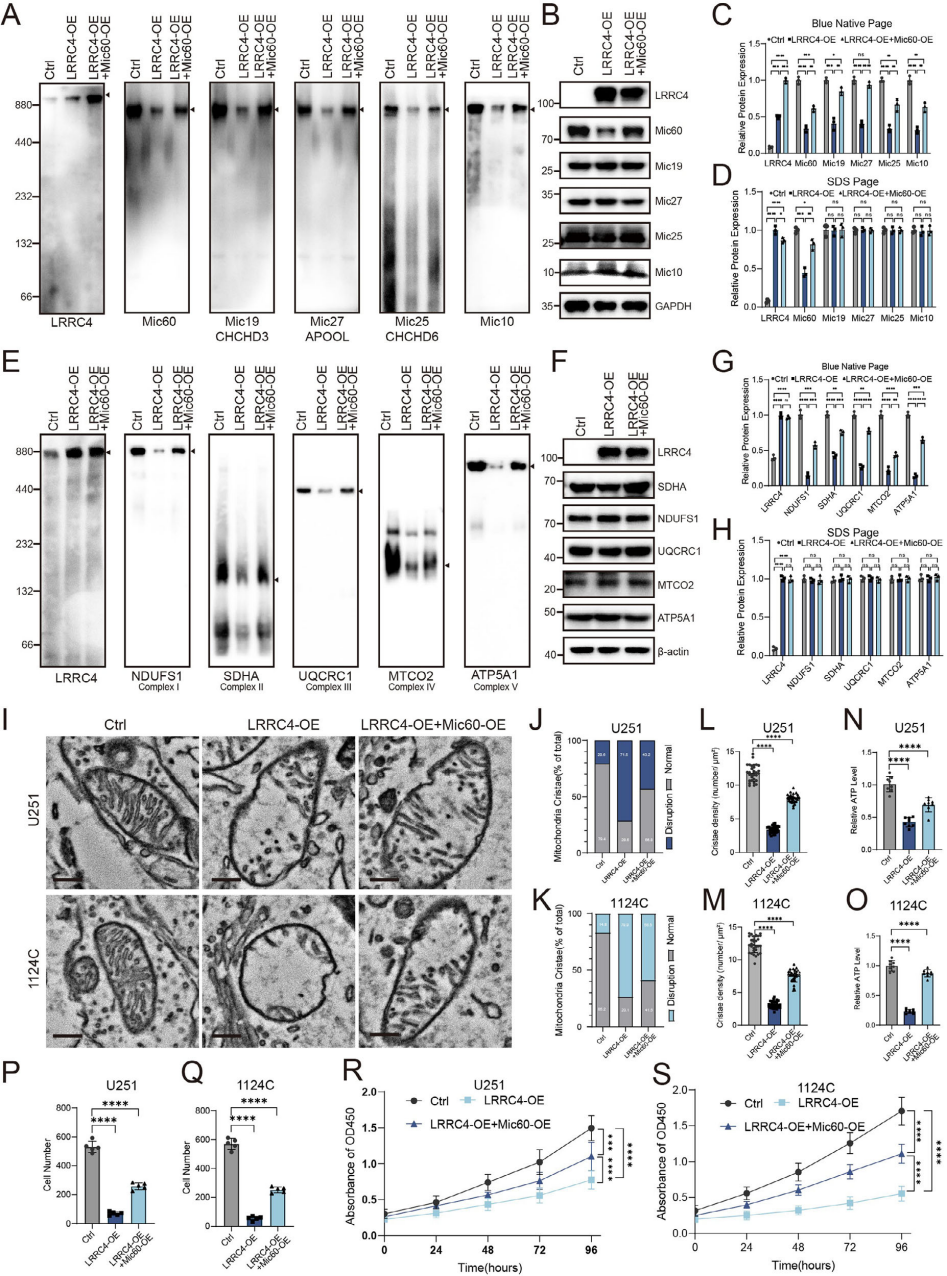
Mic60 restoration rescues LRRC4‐induced MICOS disruption, mitochondrial dysfunction, and GBM growth inhibition. (A—D) BN‐PAGE and SDS‐PAGE analyses of MICOS complex assembly and MICOS protein expression in Ctrl, LRRC4‐OE, and LRRC4‐OE + Mic60‐OE cells. LRRC4 overexpression disrupted MICOS complex assembly and reduced Mic60‐associated MICOS components, whereas Mic60 re‐expression partially restored MICOS assembly and protein levels. (E—H) BN‐PAGE and SDS‐PAGE analyses of mitochondrial respiratory chain complexes. LRRC4 overexpression impaired the native assembly of oxidative phosphorylation complexes, while Mic60 re‐expression partially rescued respiratory complex assembly. (I) Representative TEM images of mitochondria in U251 and 1124C cells under Ctrl, LRRC4‐OE, and LRRC4‐OE + Mic60‐OE conditions. Scale bars, 200 nm. (J,K) Quantification of mitochondria with normal or disrupted cristae in U251 and 1124C cells. (L,M) Quantification of mitochondrial cristae density in U251 and 1124C cells. (N,O) ATP levels in U251 and 1124C cells showing that Mic60 re‐expression partially restored LRRC4‐induced ATP reduction. (P,Q Quantification of cell number in U251 and 1124C cells under Ctrl, LRRC4‐OE, and LRRC4‐OE + Mic60‐OE conditions. (R,S) CCK‐8 proliferation assays showing that Mic60 re‐expression partially rescued the growth inhibition induced by LRRC4 overexpression in U251 and 1124C cells. Data are presented as mean ± SD from at least three independent experiments. Statistical significance was determined by one‐way ANOVA followed by Tukey's post hoc test. *p < 0.05, **p < 0.01, ***p < 0.001, ****p < 0.0001.

Next, we assessed respiratory chain organization. LRRC4 overexpression disrupted the assembly of multiple oxidative phosphorylation complexes, whereas Mic60 re‐expression partially recovered the native assembly of complexes I–V, with only modest changes in total respiratory subunit expression (Figure 7E–H). These results suggest that LRRC4 primarily impairs respiratory complex assembly via MICOS disruption.

Transmission electron microscopy revealed that the LRRC4‐overexpressing cells displayed swollen mitochondria, disrupted cristae, and reduced cristae density. Mic60 overexpression partially restored cristae organization in both U251 and 1124C cells (Figure 7I–M). Functionally, Mic60 rescue partially restored ATP levels and cell numbers that were suppressed by LRRC4 overexpression (Figure 7N–Q). Transwell invasion assays further showed that LRRC4 overexpression markedly inhibited GBM cell invasion, whereas Mic60 re‐expression partially rescued the invasive capacity of both U251 and 1124C cells (Figure 7R–S and Figure S5A).

Together, these rescue experiments demonstrated that Mic60‐centered MICOS disruption is a key causal event linking LRRC4 overexpression to cristae collapse, respiratory chain disassembly, impaired bioenergetics, and suppression of GBM cell growth and invasion.

### 2.8 LRRC4 Suppresses GBM Growth and Invasion by Downregulating AP2A1 Expression

Our previous studies have demonstrated that cytoplasmic LRRC4 inhibits GBM proliferation by interacting with diverse proteins and signal transduction pathways [24, 26, 27]. LRRC4 can also translocate to the nucleus to regulate the transcriptional repression of target genes, thereby inhibiting glioma cell migration and proliferation [28, 42, 43]. To assess the prognostic value of AP2A1, we analyzed its expression across different WHO grades in the CGGA cohort [44]. AP2A1 expression progressively increased in glioma WHO grade (II–IV) (Figure 8A), and Kaplan–Meier survival analysis revealed that patients with higher AP2A1 levels exhibited significantly poorer overall survival than those with lower levels (Figure 8B). These findings suggest that AP2A1 is a negative prognostic marker for glioma.

**FIGURE 8:**
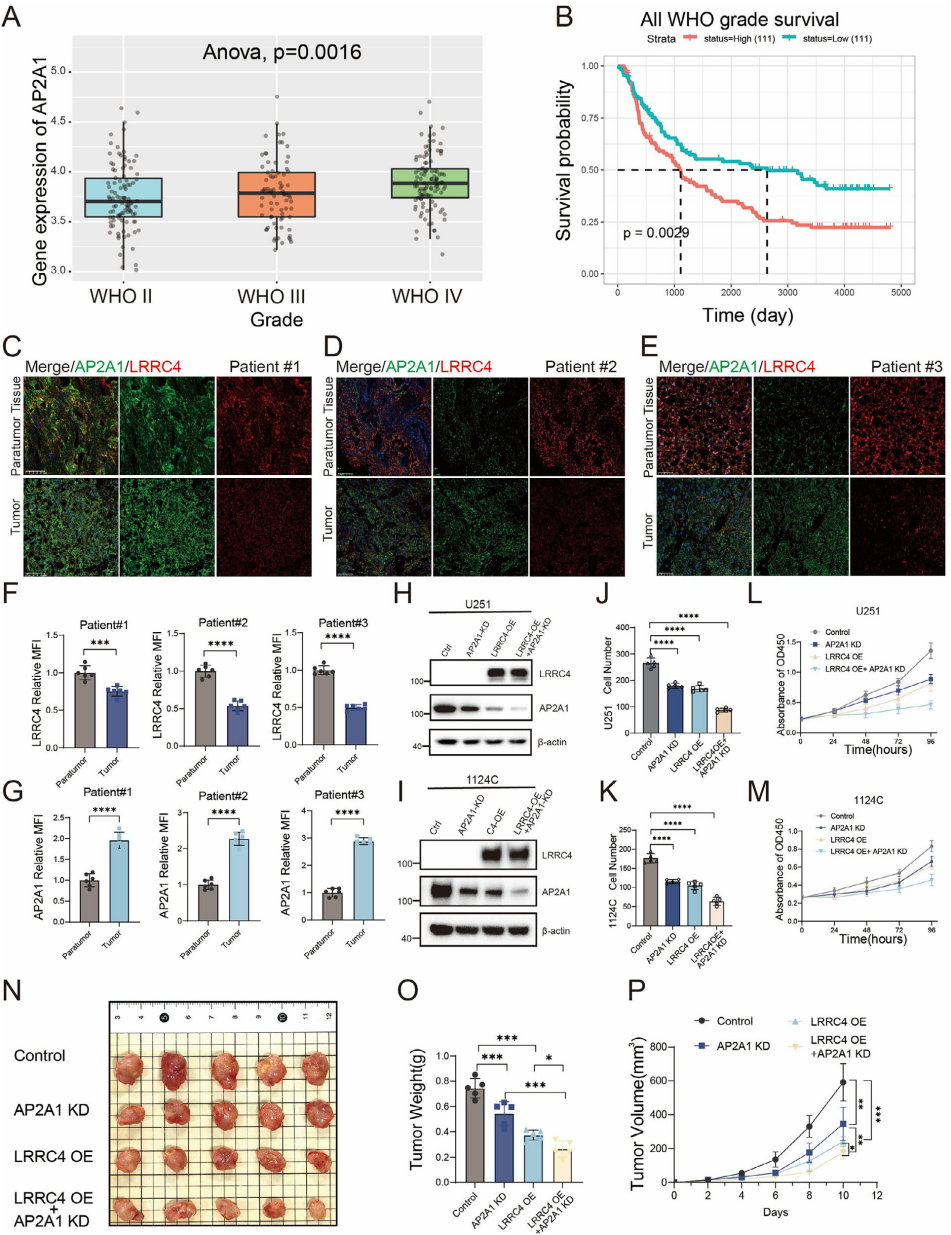
LRRC4 suppresses GBM growth by downregulating AP2A1 expression. (A) Analysis of the CGGA database (http://www.cgga.org.cn/) showed that AP2A1 expression levels increased with glioma WHO grade (ANOVA, p = 0.0016). (B) Kaplan‐Meier survival analysis revealed that glioma patients with higher AP2A1 expression had significantly worse overall survival (p = 0.0029). (C—E) Multiplex immunohistochemical staining of GBM peritumoral and tumor tissues from three patients demonstrated that LRRC4 (red) was markedly decreased, while AP2A1 (green) was strongly upregulated in tumor tissues compared with peritumoral tissues, with clear reciprocal expression patterns. (F,G) Quantification of mean fluorescence intensity (MFI) confirmed significantly lower LRRC4 (F) and higher AP2A1 (G) expression in tumor tissues compared with peritumoral tissues (n=3 patients, *** *p* < 0.001, **** *p* < 0.0001). (H,I) Western blot validation of LRRC4 and AP2A1 expression levels in U251 (H) and 1124C (I) cell lines under control, AP2A1 knockdown (KD), LRRC4 overexpression (OE), and combined conditions. (J,K) Quantitative analysis of transwell invasion assays demonstrates that the combination of LRRC4‐OE and AP2A1‐KD results in the strongest inhibition of invasive capacity in 1124C (J) and U251 (K) cells. (L‐M). CCK‐8 proliferation assays further demonstrated that LRRC4 overexpression inhibited cell proliferation, AP2A1 knockdown reversed this effect, and the combined LRRC4 overexpression plus AP2A1 knockdown condition, which reduced AP2A1 expression to the lowest level, produced the most pronounced suppression of proliferation. (N) Representative xenograft tumors from nude mice injected with control, AP2A1 knockdown, LRRC4 overexpression, or LRRC4 overexpression plus AP2A1 knockdown U251 cells. (O,P) Tumor weight(O) and tumor volume(P) analysis confirmed that LRRC4 overexpression significantly inhibited tumor growth, AP2A1 knockdown partially rescued this effect, while LRRC4 overexpression combined with AP2A1 knockdown achieved the greatest reduction in tumor burden, consistent with lowest AP2A1 expression.

Multiplex immunohistochemistry of tumor and matched peritumoral tissues from three patients revealed a reciprocal expression pattern: LRRC4 was reduced in tumor regions, whereas AP2A1 was strongly elevated (Figure 8C–E). Quantitative analysis of the mean fluorescence intensity confirmed significantly lower LRRC4 (Figure 8F) and higher AP2A1 (Figure 8G) levels in tumors versus peritumoral tissues. These data indicate a potential inverse regulatory relationship between LRRC4 and AP2A1 in GBM. To investigate the functional consequences of this relationship, U251 and 1124C cells were manipulated to overexpress LRRC4 (OE), knock down AP2A1 (KD), or combine both conditions. Western blotting confirmed the expected protein expression changes: LRRC4 OE reduced AP2A1 levels, AP2A1 KD efficiently decreased its protein expression, and the combination treatment resulted in the lowest AP2A1 abundance (Figure 8H,I and Figure S6A,B). Transwell invasion assays showed that LRRC4 OE inhibited invasion, AP2A1 KD reduced invasion, and that this combination achieved the strongest suppression in both 1124C (Figure 8J and Figure S6C) and U251 cells (Figure 8K and Figure S6C). Similarly, CCK‐8 assays demonstrated that LRRC4 OE reduced proliferation, AP2A1 KD partially rescued this effect, and the combined manipulation maximally inhibited cell growth (Figure 8L,M). Finally, xenograft experiments in nude mice confirmed these findings in vivo. Tumors from LRRC4 OE cells were markedly smaller, AP2A1 KD partially restored growth, and the combination of LRRC4 OE and AP2A1 KD yielded the smallest tumors (Figure 8N). Quantification of the tumor weight (Figure 8O) and volume (Figure 8P) validated these trends, demonstrating that LRRC4 suppresses GBM proliferation, invasion, and tumor growth by downregulating AP2A1. Simultaneous LRRC4 overexpression and AP2A1 knockdown maximizes tumor suppression, highlighting AP2A1 as a key functional mediator of LRRC4 expression in GBM.

## 3 Discussion

Mitochondrial dynamics are central to GBM proliferation, invasion, and metabolic flexibility [13, 45]. Although fission, fusion, cristae architecture, and oxidative phosphorylation have been implicated in tumor aggressiveness, the upstream mechanisms that integrate these processes remain poorly defined. Our study identified LRRC4 as a Golgi‐anchored tumor suppressor that converts vesicular trafficking into mitochondrial remodeling program. Rather than functioning solely as a signaling adaptor or membrane‐associated transcriptional regulator [31, 46], LRRC4 operates at the organelle interface, coupling Golgi‐derived clathrin‐coated vesicles to mitochondrial architecture and metabolic fitness. By integrating patient‐level transcriptomic stratification with mechanistic experiments, ultrastructural analysis, and rescue studies, we define an LRRC4–AP2A1–Mic60 axis that links Golgi‐derived clathrin‐coated vesicles to mitochondrial cristae integrity, bioenergetic capacity, and GBM growth control.

A central conceptual advance of this study was the establishment of the mitoDynamic score as a clinical‐to‐mechanistic bridge for studying mitochondrial remodeling in GBM. Mitochondrial morphology cannot be directly inferred from bulk tumor transcriptomes; however, the transcriptional activity of fission‐ and fusion‐related regulators can provide a functional proxy for mitochondrial dynamics. By applying ssGSEA to a curated mitochondrial dynamics gene set, we generated a quantitative score that stratified patients with GBM according to their mitochondrial dynamics‐related activity. This score is not merely a prognostic descriptor; it also serves as a discovery framework that connects mitochondrial dynamics to patient survival, molecular subtype, IDH1 status, and candidate upstream regulators. Using this approach, LRRC4 has emerged as a clinically relevant regulator associated with preserved mitochondrial dynamics and favorable outcomes. Thus, the mitoDynamic score enabled us to move from a patient‐derived mitochondrial dynamics signature to the experimental identification of a Golgi‐localized tumor suppressor that controls mitochondrial architecture. The mitoDynamic score reinforces the clinical relevance of this pathway. Analysis of patient tumors revealed that low LRRC4 expression coupled with high AP2A1 expression was associated with poor survival, whereas higher LRRC4 levels were correlated with enhanced mitochondrial dynamics and improved clinical outcomes. Consistent with these observations, in vitro experiments demonstrated that LRRC4 overexpression or AP2A1 knockdown individually reduced GBM cell proliferation and invasion; However, the combination of LRRC4 overexpression and AP2A1 knockdown produced the most pronounced suppression. Similarly, in xenograft models, tumors exhibited maximal growth inhibition under the combined conditions, reinforcing the functional importance of the LRRC4–AP2A1 axis. These results link patient‐derived transcriptional signatures, mitochondrial dynamics, and experimental validation, illustrating how organelle‐level regulation translates into tumor phenotypes. Moreover, the mitoDynamic score provides a clinically actionable framework to identify not only LRRC4, but also other candidate genes that regulate mitochondrial dynamics and tumor progression, offering a systematic approach to uncover additional regulators of organelle architecture in GBM.

Our study also shows that LRRC4 cooperates with Golgi‐derived AP2A1‐containing clathrin‐coated vesicles to modulate mitochondrial fission and fusion. AP2A1 encodes the α1‐adaptin subunit of the AP‐2 complex, which is a clathrin adaptor involved in vesicle formation and endocytic trafficking [47]. While previous studies have suggested that Golgi vesicles facilitate Drp1‐mediated mitochondrial scission at ER–mitochondria contact sites [39], our findings reveal a distinct Golgi mitochondria crosstalk and a tumor‐suppressive mechanism. LRRC4 directly binds to AP2A1, promotes its ubiquitination‐mediated degradation, and redistributes AP2A1 between the cytosolic and mitochondrial fractions, thereby remodeling the availability and localization of AP2A1‐associated vesicles. AP2A1 knockdown phenocopies the LRRC4‐induced mitochondrial elongation and fusion‐like morphology, accompanied by reduced levels of fission mediators and increased levels of fusion‐associated proteins, confirming its critical role in maintaining mitochondrial fission in GBM cells [47]. At the mechanistic level, LRRC4 repurposes AP2A1‐containing vesicles from a canonical trafficking function to a tumor‐suppressive mitochondrial remodeling program. This is the first evidence that Golgi‐derived clathrin‐coated vesicles act as active modulators of mitochondrial dynamics, converting clathrin‐coated vesicles into a tumor‐suppressive structural checkpoint. Recent advances have highlighted that mitochondrial fission is not solely orchestrated by ER and actin cytoskeleton but also involves other membrane‐bound organelles such as lysosomes and Golgi‐derived vesicles [20, 39]. Our findings extend this concept by demonstrating that LRRC4 actively redirects AP2A1‐coated Golgi vesicles to penetrate or intimately fuse with the mitochondrial inner membrane, as evidenced by high‐resolution mitochondrial substructure purification and Proteinase K protection assays. This indicates a non‐canonical topological interaction, distinct from classical OMM fission models, where TGN‐derived PI(4)P vesicles or VPS13B deliver lipids and provide mechanical support for Drp1‐mediated cleavage [23, 39]. Such organelle‐level crosstalk directly regulates cristae integrity, fission‐fusion balance, and respiratory chain assembly, revealing that perturbations in Golgi–mitochondrial communication can profoundly alter tumor bioenergetics and proliferation.

The downstream mitochondrial target of this pathway is the MICOS complex, with the Mic60‐centered subcomplex acting as the central structural effector. The MICOS complex, particularly Mic60, serves as a central structural effector downstream of LRRC4‐AP2A1 signaling. The MICOS complex is composed of an MIC60‐containing subcomplex (MIC60‐MIC25‐MIC19) that stabilizes contact sites between the inner and outer membranes, and an MIC10‐containing subcomplex (MIC10‐MIC13‐MIC26‐MIC27) that shapes the cristae membranes [48, 49, 50, 51]. Both are essential for the integrity of the cristae junction and for efficient oxidative phosphorylation [48, 49, 50, 51]. LRRC4 overexpression disrupts MICOS assembly, reduces Mic60 levels, fragments cristae, and destabilizes respiratory chain complexes, leading to impaired mitochondrial membrane potential, reduced ATP production, and suppressed TCA cycle and nucleotide biosynthesis. Mic60 reexpression in LRRC4‐overexpressing cells partially restored MICOS assembly, cristae integrity, respiratory complex organization, ATP levels, and invasive capacity, providing direct evidence that MICOS disruption is a primary consequence of LRRC4 activity rather than a secondary effect of mitochondrial stress. This partial rescue further indicates that additional vesicle‐associated cargoes, mitochondrial membrane proteins, or contact‐site components may cooperate with MICOS to fully execute the tumor‐suppressive phenotype. These observations establish a hierarchical mechanistic sequence whereby LRRC4 engages AP2A1‐containing clathrin‐coated vesicles, redirects them to the mitochondria, disrupts MICOS/Mic60, collapses cristae, destabilizes respiratory complexes, and compromises metabolic output. Beyond structural perturbations, LRRC4‐induced MICOS disruption triggers metabolic reprogramming; GBM cells rely on glycolysis under normoxia yet retain oxidative phosphorylation flexibility [45, 52, 53]. Loss of crista integrity and respiratory chain destabilization impair ATP production, reduce ROS buffering, and limit anabolic metabolism, creating a checkpoint that constrains tumor growth and invasion. Importantly, these findings revealed a previously uncharacterized interface between Golgi‐derived clathrin‐coated vesicles and mitochondrial inner membrane organization, demonstrating how organelle crosstalk integrates structural remodeling with metabolic control to regulate GBM aggressiveness and highlighting the therapeutic potential of targeting Golgi‐derived vesicle‐mediated mitochondrial architecture.

This study had several limitations. While the mitoDynamic score captures transcriptional proxies of mitochondrial dynamics, it does not provide single‐cell morphological resolution or directly measure vesicle–mitochondria contacts. However, the mechanism through which LRRC4 reduces Mic60 expression remains unclear.

In conclusion, this study uncovered a novel Golgi‐to‐mitochondrial tumor‐suppressive axis in GBM, in which LRRC4 coordinates AP2A1‐containing clathrin‐coated vesicle trafficking to the mitochondria, leading to disassembly of the Mic60‐centered MICOS scaffold. This inter‐organelle communication disrupts crista architecture, destabilizes respiratory chain supercomplexes, and imposes metabolic constraints that collectively suppress proliferation and invasion. Our findings provide the first direct evidence that Golgi‐derived clathrin‐coated vesicles function as active modulators of mitochondrial dynamics by converting the classical trafficking pathway into a tumor‐suppressive mitochondrial remodeling mechanism. By integrating patient‐derived mitoDynamic scoring with mechanistic rescue experiments, we provided a systematic framework for identifying the regulators of mitochondrial dynamics in GBM and established the LRRC4–AP2A1–Mic60 axis as a mechanistically and clinically validated vulnerability. These results not only advance our understanding of Golgi mitochondria crosstalk in tumor suppression but also suggest that targeting vesicles derived from membrane‐bound organelles that are trafficked to mitochondria may represent a novel therapeutic strategy for GBM.

## 4 Materials and Methods

### 4.1 Establishment of the mitoDynamic Score and Survival Analysis

A mitoDynamic gene set was obtained from published literature describing key regulators of mitochondrial dynamics [14]. Transcriptomic and clinical data of glioblastoma (GBM) patients were downloaded from The Cancer Genome Atlas (TCGA). The mitoDynamic score for each sample was calculated using the single‐sample Gene Set Enrichment Analysis (ssGSEA) algorithm implemented in the GSVA package (version 1.46.0; Hänzelmann et al., 2013). This score serves as a quantitative metric of the activity of mitochondrial dynamics‐related genes; a higher score indicates greater enrichment or dysregulation of these processes, which is often associated with metabolic stress and increased tumor aggressiveness.

Patients were dichotomized into high and low groups using surv_cutpoint (survminer v0.4.9) and analyzed by Kaplan‐Meier and log‐rank tests [54]. This method utilizes maximally selected rank statistics to identify the threshold that provides the most significant separation in overall survival (OS) between the two groups, thereby ensuring an objective and statistically rigorous grouping. Kaplan‐Meier survival analysis and the log‐rank test were used to assess prognostic significance. Associations between mitoDynamic score and clinical or molecular features (including transcriptomic subtypes and IDH1 mutation status) were evaluated using the Wilcoxon test. Univariate and multivariate Cox regression analyses were conducted to determine the independent prognostic value of the mitoDynamic score. Differentially expressed genes (DEGs) between high and low score groups were identified using the “limma” package, and results were visualized with volcano plots.

### 4.2 Clinical Data Stratification

For clinical validation, mRNA sequencing data and matched survival information were obtained from the Chinese Glioma Genome Atlas (CGGA, http://www.cgga.org.cn). Patients within each cohort (mRNAseq_693, mRNAseq_325) were categorized into high‐ and low‐expression groups using the median mRNA expression value of AP2A1 as the cutoff point.

### 4.3 Construction of plV2‐LRRC4‐FLAG‐His Expression Vector

The pLV2‐LRRC4‐3FLAG‐12His expression vector was constructed by cloning the full‐length human LRRC4 coding sequence (CDS, NM_022143.5) into a linearized pLV2 backbone. The full‐length LRRC4 CDS (devoid of the natural stop codon) was amplified from human U251 cDNA using high‐fidelity DNA polymerase. A C‐terminal 3FLAG‐12His tag sequence was strategically introduced into the reverse primer, and 25 bp homologous overlaps with the linearized pLV2 vector were incorporated into both primers to facilitate seamless assembly. The following primers were designed for LRRC4 amplification and in‐frame fusion: F‐LRRC4 F‐TGTCTCATCATTTTGGCAAAGCTCTTGTGGCAGGTAACTGTGCAC, R:GTGATGATGGTGGTGGTGATGATGGTGGTGGTGATGGCTTGTCATCGTCATCCTTGTAATCGATGTCATGATCTTTATAATCACCGTCATGGTCTTTGTAGTCATTTGAGTTTCCTGTACCTTGTCCTT. The purified PCR products and the linearized vector were assembled using the DNA Assembly Mix Ultra (BestEnzymes Biotech, EG24204S) at 50°C for 60 min. The resulting constructs were transformed into DH5α competent cells, and positive clones were identified by colony PCR and subsequently verified by Sanger sequencing (Tsingke, China) to confirm sequence fidelity and correct reading frame maintenance. The validated pLV2‐LRRC4‐FLAG‐His construct was employed for subsequent transient transfections and functional characterization assays.

### 4.4 Tissue samples

GBM samples and peritumoral brain tissue were obtained from patients undergoing surgery at the Department of Neurosurgery, Xiangya Hospital. All protocols were reviewed and approved by the Joint Ethics Committee of the Central South University Health Authority (Ethics Protocol No. 2024030156, reviewed and approved on March 5, 2024).

### 4.5 Cell Culture and Generation of Stable Cell Lines

GBM cells (U251 cell, 1124C cell) and glial cells (HEB) were obtained from the Cancer Research Institute of Central South University. Cells were maintained in dulbecco's modified eagle medium(DEME) at 37°C and 5% CO2 in Dulbecco's modified Eagle's medium (DMEM; C11995500BT, HyClone) supplemented with 10% fetal bovine serum and antibiotics (P1400, Solarbio). Lentiviral particles were produced in HEK‐293T cells (2 × 10^6^ cells/dish) by co‐transfecting the pLV2 vector, psPAX2, and pMD2.G (4 µg DNA total) using PEI MAX 40K (24765‐1, Polyscience) in 150 mM NaCl. Supernatants were harvested at 60 h, filtered (0.45 µm), and used for double infection of GBM cells (20%–30% density) with 8 µg/ml polybrene (sc‐134220, SCBT). Stable populations were established via 2 µg/ml puromycin selection starting 48 h post‐transduction. For all subsequent functional and biochemical experiments, the expression of LRRC4‐FLAG‐His and control vector (Flag‐His) were induced by adding 2 µg/mL doxycycline (Dox) to the culture medium for 48 h prior to harvesting.

### 4.6 Immunofluorescence and Immunohistochemistry

U251 and 1124C cells were seeded in six‐well plates, with two coverslips placed in each well, and cultured for 48 h at 37°C. Cells were incubated with 100 nM MitoTracker Red CMXRos (M7512, Invitrogen) at 37°C for 30 min, followed by fixation in 4% paraformaldehyde at room temperature for 10 min. Permeabilization was carried out using 0.5% Triton X‐100 for 10 min at room temperature. The slides were then washed and blocked with 1% BSA for 1 h. U251 and 1124C cells were incubated with primary antibodies overnight at 4°C. The following morning, the slides were incubated with Alexa Fluor 488 (A11034, Abcam) and Alexa Fluor 647(A21236, Abcam) for 1 h at room temperature. Cell nuclei were stained with DAPI (C1002, Boyetime). Immunofluorescence images were captured using a Zeiss LSM 980 confocal microscope and analyzed with ImageJ software. Mitochondrial morphology was quantitatively analyzed using ImageJ (Fiji distribution, v1.53c) with the Mitochondrial Network Analysis (MiNA) toolset, as described previously [55].

For the quantification of fluorescence intensity specifically within the mitochondrial compartment, the MitoTracker Red channel was processed to generate a binary mask through global thresholding. This mask defined the mitochondrial Region of Interest (ROI), which was subsequently mapped onto the secondary channels (Alexa Fluor 488/FLAG or Alexa Fluor 647/AP2A1) using the ROI Manager tool. The Mean Fluorescence Intensity (MFI) was calculated for each mitochondrial ROI, with background correction performed by subtracting the average signal from three randomly selected cell‐free regions per field of view.

### 4.7 Co‐Immunoprecipitation

GBM cells transfected with LRRC4‐FLAG were prepared and lysed in precooled RIPA lysis buffer supplemented protease inhibitor cocktail (B14001, Biomake). Cell lysates were incubated overnight at 4°C with an anti‐Flag antibody (F1804, Sigma). The mixtures were then incubated with Protein A/G Magnetic Beads (B23202, Bimake) for an additional 12 h at 4°C. Following three washes, the beads were boiled in 1× SDS loading buffer. The immunoprecipitated proteins were subsequently analyzed by Western blotting using an anti‐AP2A1 antibody (29887‐1‐AP, Proteintech).

### 4.8 RNA Sequencing

Total RNA was harvested using the TRIzol method (Invitrogen), quality verified with NanoDrop 2000 spectrophotometer, structural integrity was confirmed using an Agilent 2100 Bioanalyzer. Libraries were constructed via the VAHTS Universal V6 RNA‐seq kit and subjected to deep sequencing on the Illumina NovaSeq 6000 system, generating 150 bp paired‐end reads. Raw data filtration was executed through fastp software to obtain high‐quality clean reads for downstream alignment. Mapping to the human reference genome (GRCh38) was performed using HISAT2, and transcript abundance was determined as FPKM, with read counts derived via HTSeq‐count. PCA was employed to verify sample consistency across biological replicates. Differentially expressed genes (DEGs) were identified using the DESeq2 package (q‐value < 0.05; log2| (FoldChange)| > 1) and visualized through hierarchical clustering. Functional enrichment analysis, including GO, KEGG, and GSEA, was performed to delineate shifts in metabolic circuitries, specifically focusing on the TCA cycle and OXPHOS signatures.

### 4.9 Targeted Energy Metabolomics

Cell pellets were homogenized in 200 µL of pre‐chilled 80% methanol supplemented with 10 µL of 10 mM Succinic acid‐D6 as an internal standard. Chloroform (400 µL) and deionized water (100 µL) were added, followed by vortexing and centrifugation at 14 000 rcf for 20 min at 10°C. The aqueous polar fraction (200 µL) was vacuum‐dried and reconstituted in an acetonitrile/water mixture (1:1, v/v). Chromatographic separation was achieved on an Agilent 1290 Infinity II UPLC system utilizing a Waters ACQUITY UPLC BEH Amide column (1.7 µm, 2.1 mm × 150 mm) at 35°C. The mobile phase comprised 50 mM ammonium acetate with 1.2% ammonium hydroxide (Phase A) and 1% acetylacetone in acetonitrile (Phase B) at a flow rate of 300 µL/min. Absolute quantification was conducted on a Sciex 5500 QTRAP mass spectrometer in multiple reaction monitoring (MRM) mode under negative electrospray ionization (ESI‐). Data were analyzed using MultiQuant 3.0.2 software, and significant differential metabolites were screened based on a p‐value < 0.05 and Fold Change thresholds.

### 4.10 Proteomic Analysis

U251 cells with pLV2‐LRRC4‐FLAG‐His Vector were prepared in 10 cm dishes, induced with 2 µg/ml doxycycline (DOX) for 48 h, and harvested at 80% confluence to ensure maximum experimental consistency. Whole‐cell protein lysates were subjected to Data‐Independent Acquisition (DIA) proteomics. Extracted proteins were reduced with DTT, alkylated with iodoacetamide, and digested overnight with sequencing‐grade trypsin. Peptide fractions were analyzed on an Orbitrap Fusion Lumos mass spectrometer. Protein identification and relative quantification were performed using Spectronaut software (Biognosys), employing a dynamic mass tolerance strategy. Differentially expressed proteins (DEPs) were filtered based on a fold‐change threshold of 1.5 and an adjusted p‐value < 0.05. Mitochondrial proteins were further refined by intersection with the MitoCarta 3.0 inventory to examine the stability of the MICOS complex.

### 4.11 siRNA Transfection

Transient silencing of target genes was performed using Lipofectamine 3000 (L3000015, Invitrogen) according to the manufacturer's instructions. Specifically, GBM cells were seeded into 6‐well plates and transfected at 70% confluence with 50 nM of siRNA targeting human AP2A1 (siRNA#1: 5’‐ GCTACAGTAAGAAAAAATA ‐3’ siRNA#1: 5’‐ GGAGACAAAGCCTTGGATG‐3’) or a non‐targeting control siRNA(5’‐UUCUCCGAACGUGUCACGU‐3’). Cells were harvested 72 h post‐transfection for downstream functional assays. Knockdown efficiency was rigorously validated at both transcript and protein levels via Western blotting before proceeding with mitochondrial morphology analysis.

### 4.12 Mitochondria Purification and Proteinase K Protection Assay

When GBM cells have grown to confluence in three 15 cm dishes, the cells were scraped and transferred into 15 mL centrifuge tubes. These tubes were then centrifuged at 800×g for 5 min at room temperature, and the supernatant was discarded. The cell pellet was resuspended in dulbecco's phosphate buffered saline (DPBS), washed, and centrifuged again under the same conditions. Subsequently, the pellet was resuspended in 5.5 mL of 1× MS Homogenization Buffer (ab288084, Abcam) and transferred to a 7 mL KIMBLE Dounce tissue grinder set (D9063, Sigma). Homogenization was performed using pestle A for 100 passes. The resulting homogenate was then transferred to a 15 mL centrifuge tube. Low‐speed centrifugation was performed at 1300 × g for 5 min at 4°C to remove cell debris, nuclei, and unbroken cells. Following this, 100 µL of the supernatant was collected, and the pellet was resuspended in 1.5 mL of Homogenization Buffer. The supernatant was aspirated, and the low‐speed centrifugation was repeated twice more under the same conditions. For high‐speed centrifugation, the sample was transferred to a new centrifuge tube and centrifuged at maximum speed at 4°C for 15 min. Finally, 100 µL of the supernatant was collected, and the remaining supernatant was discarded to obtain the mitochondrial pellet.

Purified mitochondria (20 µg) were resuspended in MS buffer and incubated with varying concentrations of digitonin (0%–2%) and Proteinase K (100 µg/mL) for 15 min at 4°C. A sample treated with 1% Triton X‐100 served as a control for complete membrane permeabilization. Proteinase K activity was inhibited by adding 2 mM PMSF for 15 min at 4°C. Mitochondria were subsequently lysed with 4× SDS sample buffer and heated at 95°C for 15 min before SDS‐PAGE and immunoblotting. The integrity and submitochondrial localization of proteins were assessed using markers for outer membrane (Tom20), inner membrane (TIM23, Mic60), and control proteins (e.g., Cep89, COX IV).

### 4.13 Golgi‐Apparatus Purification

To isolate the Golgi apparatus using the Golgi Apparatus Extraction Kit (EX1360, Solarbio), start by harvesting 2 × 10^7 GBM cells and centrifuging them at 500 × g for 3 min at 4°C. Following the aspiration of the culture medium, the cells were washed twice with cold PBS to remove extracellular debris. The resulting cell pellet was resuspended in 500 µL of chilled lysis reagent and incubated on ice for 10 min. Mechanical homogenization was conducted using a Dounce grinder for 5 min to ensure cellular disruption. The homogenate was then subjected to sequential centrifugation steps: initially at 1000 × g for 5 min, followed by 3000 × g for 10 min, and finally at 5000 × g for 10 min at 4°C, with the supernatant collected after each interval. To isolate the Golgi membranes, 10 µL of a specific binding reagent was added to the supernatant, which was subsequently centrifuged at 20 000 × g for 20 min. The crude Golgi pellet was resuspended in 500 µL of cold preservation reagent and underwent a final purification centrifugation at 20 000 × g for 30 min at 4°C. The final purified Golgi fraction was resuspended in preservation solution for biochemical analysis.

### 4.14 Immunoblotting

GBM cells and HEB were lysed using 500 µL of 4× SDS loading Sample buffer containing 1 mM phosphatase inhibitor and 1 mM phenylmethylsulfonyl fluoride (PMSF). Denatured proteins (20 µg) were resolved on 10% SDS‐PAGE gels and transferred to 0.45 µM PVDF membranes (Thermo Fisher). After blocking with 5% milk at room temperature for 1 h, the membranes were incubated with the primary antibody overnight at 4°C, followed by a 2‐h incubation with a horseradish peroxidase‐conjugated secondary antibody at room temperature. After washing the membranes three times with TBST, chemiluminescent detection was performed after incubate with ECL Reagent (Sangon Biotech, China). Following antibodies were used: LRRC4(HPA051100, Atlas); FLAG (F‐1804, Sigma); AP2A1 antibody (29887‐1‐AP, Proteintech), Drp1(12957‐1‐AP, Proteintech), OPA1(27733‐1‐AP, Proteintech), MFN1(13798‐1‐AP, Proteintech), OMA1(bs‐19641R, Bioss), UQCRC1(D123504, Sangon Biotech), NDUFS1(D122747, Sangon Biotech), SDHA(14865‐1‐AP, Proteintech), MTCO2(55070‐1‐AP, Proteintech), ATP5A1(14676‐1‐AP, Proteintech), GORASP2(P31596, ProMabBiotechnologies Inc), MIC25(20639‐1‐AP, Proteintech), MIC10(31561‐1‐AP, Proteintech), MIC19(25625‐1‐AP, Proteintech), MIC27 (28514‐1‐AP, Proteintech), MIC60 (10179‐1‐AP, Proteintech), GAPDH (60004‐1‐Ig, Proteintech),β‐Act (66009‐1‐Ig, Proteintech), α‐Tub(P42959, ProMabBiotechnologies Inc).

### 4.15 Blue‐Native PAGE Analysis

Native protein complexes were analyzed using Blue Native PAGE (BN‐PAGE). GBM cells were harvested from 10 cm culture dishes and collected via centrifugation at 800 rpm for 5 min. Cell pellets were resuspended in 200 µL of pre‐chilled digitonin‐buffer (1% digitonin, 20 mM Tris‐HCl pH 7.4, 0.1 mM EDTA, 50 mM NaCl, 10% glycerol, 1 mM PMSF, and 1 mM PI) and incubated on ice for 30 min to facilitate membrane solubilization while preserving protein‐protein interactions. Lysates were centrifuged at maximum speed for 15 min at 4°C and 100 µL of the supernatants were collected for protein quantification. Electrophoresis was performed using gradient gels, with the inner chamber filled with cathode buffer containing Coomassie G250 to impart a negative charge to the native complexes. The run was initiated at 600 V; once the complexes entered the separation gel, the cathode buffer was replaced with a Coomassie‐free version, and the electrophoresis was continued for a total of 6 h. Following separation, the native complexes were transferred to PVDF membranes for immunoblotting against respiratory chain and MICOS subunits. Following separation, protein complexes were transferred to a PVDF membrane and blocked with 5% skim milk for 60 min. Then, the PVDF membrane was incubate with the primary antibody overnight at 4°C. After wash three times with 1× PBST, then incubate the PVDF membrane with the secondary antibody at room temperature for 1 h, wash again with 1× PBST three times and chemiluminescence was captured by X‐ray films Chemidoc imaging system (Bio‐Rad).

### 4.16 Cell Proliferation and Invasion Assay

U251 and 1124C cells were added in 96‐well plates at a density of 5000 cells per well, cell viability was assessed using the Cell Counting Kit‐8(96992, Sigma‐Aldrich) following the manufacturer's instructions. U251 and 1124C cells were seeded into the upper chambers of 24‐well transwell plates (3422, Corning) at a density of 2.5 × 10^4^ cells in 300 µl of serum‐free medium. The lower chambers were filled with 500 µl of medium containing 20% FBS. The cell migration assay was then conducted according to the manufacturer's instructions.

### 4.17 Flow Cytometry

To assess mitochondrial physiological status, cells were dissociated and stained with fluorescent probes. Mitochondrial membrane potential (MMP) was determined by incubating cells with 100 nM MitoTracker Red CMXRos (Invitrogen) for 30 min at 37°C. For mitochondrial reactive oxygen species (mROS) detection, cells were loaded with 5 µM MitoSOX Red (Invitrogen) for 15 min. After two washes with pre‐warmed DPBS, fluorescence signatures were captured using a BD CytoFLEX flow cytometer. A minimum of 10 000 gated events were analyzed per sample using FlowJo V10 software to ensure statistical robustness, and data were represented as mean fluorescence intensity (MFI) normalized to control groups.

### 4.18 Measurement of Intracellular ATP

Intracellular ATP levels were quantified using the Enhanced ATP Assay Kit (AKOP004M, Beijing Boxbio Science & Technology Co.,Ltd). The cultural medium was removed from 6‐well plates, and cells were lysed on ice by adding 200 µL of lysis buffer to each well. The resulting cell lysates were collected and centrifuged at 12 000 × g for 5 min at 4°C, and the supernatants were retained for analysis. The ATP detection working solution was prepared by diluting the assay reagent with diluent at a 1:4 ratio. Aliquots of 100 µL of this working solution were added to the wells and allowed to stabilize for 5 min at room temperature to minimize background signal. Following the addition of 2 µL of each sample, relative light units (RLU) were measured using a luminometer, and ATP concentrations were calculated based on a standard curve [28].

### 4.19 Seahorse Assay

To assess mitochondrial function and metabolic reprogramming in GBM cells, a Seahorse XF Analyzer (Agilent Technologies) was used to measure the oxygen consumption rate (OCR) and extracellular acidification rate (ECAR) according to the manufacturer's instructions. GBM cells with different LRRC4 expression levels were seeded into Seahorse XF96 cell culture plates at a density of 20 000 cells per well and incubated overnight to allow cell attachment. Prior to the assay, the culture medium was replaced with Seahorse XF Base Medium supplemented with glucose (10 mM), pyruvate (1 mM), and L‐glutamine (2 mM), and the cells were equilibrated in a non‐CO_2_ incubator for 1 h at 37°C. For mitochondrial stress testing, sequential injections of oligomycin (1 µM), carbonyl cyanide 4‐(trifluoromethoxy)phenylhydrazone(FCCP, 1 µM), and rotenone/antimycin A (0.5 µM) were performed to evaluate basal respiration, ATP production, maximal respiratory capacity, and non‐mitochondrial respiration. ECAR measurements were conducted using the glycolytic stress test with injections of glucose (10 mM), oligomycin (1 µM), and 2‐deoxy‐D‐glucose (50 mM) to assess glycolytic activity. The data were analyzed to determine mitochondrial respiratory chain activity, glycolysis, and overall metabolic reprogramming.

### 4.20 Transmission Electron Microscopy and Mitochondrial Morphometric Quantification

Adherent cells were sequentially processed to preserve ultrastructural integrity. Briefly, cells were fixed in situ with 2.5% glutaraldehyde in 0.1 M phosphate buffer (pH 7.4) for 2 h at room temperature, followed by post‐fixation in 1% osmium tetroxide for 1.5 h. After graded dehydration in ethanol and infiltration with Epon 812 resin, polymerization was conducted at 60°C for 48 h. Ultrathin sections (75 nm) were obtained using a Leica EM UC7 ultramicrotome and counterstained with uranyl acetate and lead citrate. Micrographs were acquired using a Hitachi H‐7650 transmission electron microscope. Mitochondrial cristae rupture was quantified by calculating the percentage of mitochondria with broken cristae from TEM images (20 000× magnification; scale bar 500 nm). For each condition, ≥50 mitochondria from 10 randomly selected cells were analyzed. Mitochondria with disrupted or fragmented cristae were counted as damaged.

Mitochondrial ultrastructure in 1124C and U251 cells was analyzed using Transmission Electron Microscopy (TEM) and ImageJ software. After scale calibration, the OMM of each organelle was manually traced to calculate the sectional area (A) and perimeter (P). Mitochondrial area (µm^2^), aspect ratio (AR = long/short axis), cristae density (number/µm^2^) were measured using ImageJ software [56]. To ensure objectivity, all quantifications were performed by three independent researchers in a randomized and blinded manner, with at least 30 mitochondria analyzed per group. Statistical significance was determined by Student's *t*‐test, with *p* < 0.05 considered significant.

### 4.21 Xenograft Mouse Models

In vivo tumor progression was evaluated in 5‐week‐old female BALB/c nude mice. All animal experiments were performed under the approval of the Ethics Committee of Xiangya Hospital (No. 2024030156). Stably transfected U251 cells (5 × 10^6^ cells per mouse) were resuspended in 100 µL of a PBS/Matrigel mixture (1:1 ratio) and subcutaneously injected into the right flank. Tumor dimensions were monitored every 3 days using digital calipers, and volumes were calculated using the formula: V  =  (Length  ×  Width2) / 2. At 4 weeks post‐injection, mice were humanely euthanized, and the excised tumors were weighed.

### 4.22 Statistical Analyses

All quantitative results and representative images in this study were derived from at least three biologically independent replicates to ensure the reproducibility of the findings. Quantitative results from in vitro experiments, including Seahorse flux analysis (n = 5 per group) and metabolomic profiling (n = 6 per group), were presented as the mean ± standard deviation (SD) or mean ± standard error of the mean (SEM). For the analysis of mitochondrial morphology, at least 20 cells per group were randomly selected for morphometric quantification using ImageJ software. Mean fluorescence intensity (MFI) for immunofluorescence data was also calculated using ImageJ across at least 6 biological replicates.

## Author Contributions

**Yang Li**: Conceptualized the study, conducted experiments, performed data analysis, provided project funding, and wrote the manuscript; **Cheng Huang**: Assisted in experimental design, provided technical support and project funding; **Liangqi Jiang**: Assisted with experiments, contributed to bioinformatics analyses, and contributed to data interpretation; **Zhen Li**: Contributed to experiment design, data validation, and manuscript revisions; **Qing Liu**: Supervised the study, provided project funding, and critically reviewed the manuscript (Corresponding Author); **Minghua Wu**: Supervised the study, provided conceptual input, and contributed to manuscript revisions (Corresponding Author).

## Funding

This study was Project supported by the National Natural Science Foundation of China (Grant No. 82403075, No. 82172834) and the Natural Science Foundation of Hunan Province (Grant No. 2025JJ60498).

## Ethics Statement

The study was conducted in accordance with the Declaration of Helsinki, and approved by the Ethics Committee of Xiangya Hospital (2024030156). Written informed consent has been obtained from the patients to publish this paper.

## Consent

All authors agree to publish.

## Conflicts of Interest

The authors declare that they have no competing interests.

## Supporting information




**Supporting File 1**: advs76465‐sup‐0001‐SuppMat.docx.

**Supporting File 2**: advs76465‐sup‐0002‐TableS1.xlsx.

**Supporting File 3**: advs76465‐sup‐0003‐TableS2.xlsx.

**Supporting File 4**: advs76465‐sup‐0004‐TableS3.xlsx.

## Data Availability

The datasets used and/or analyzed during the current study are available from the corresponding author on reasonable request.
