# Supplementary material for: LRRC4 Orchestrates AP2A1‐Containing Clathrin‐Coated Vesicles to Disrupt Mitochondrial Cristae and Restrict Glioblastoma Progression
Source: Adv Sci (Weinh). 2026 Jul 13:e76465. Online ahead of print. doi: 10.1002/advs.76465 (PMC13360104; doi:10.1002/advs.76465)
Supplement: Supplementary file 1 — Supporting File 1: advs76465‐sup‐0001‐SuppMat.docx. [file ADVS-9999-e76465-s003.docx]

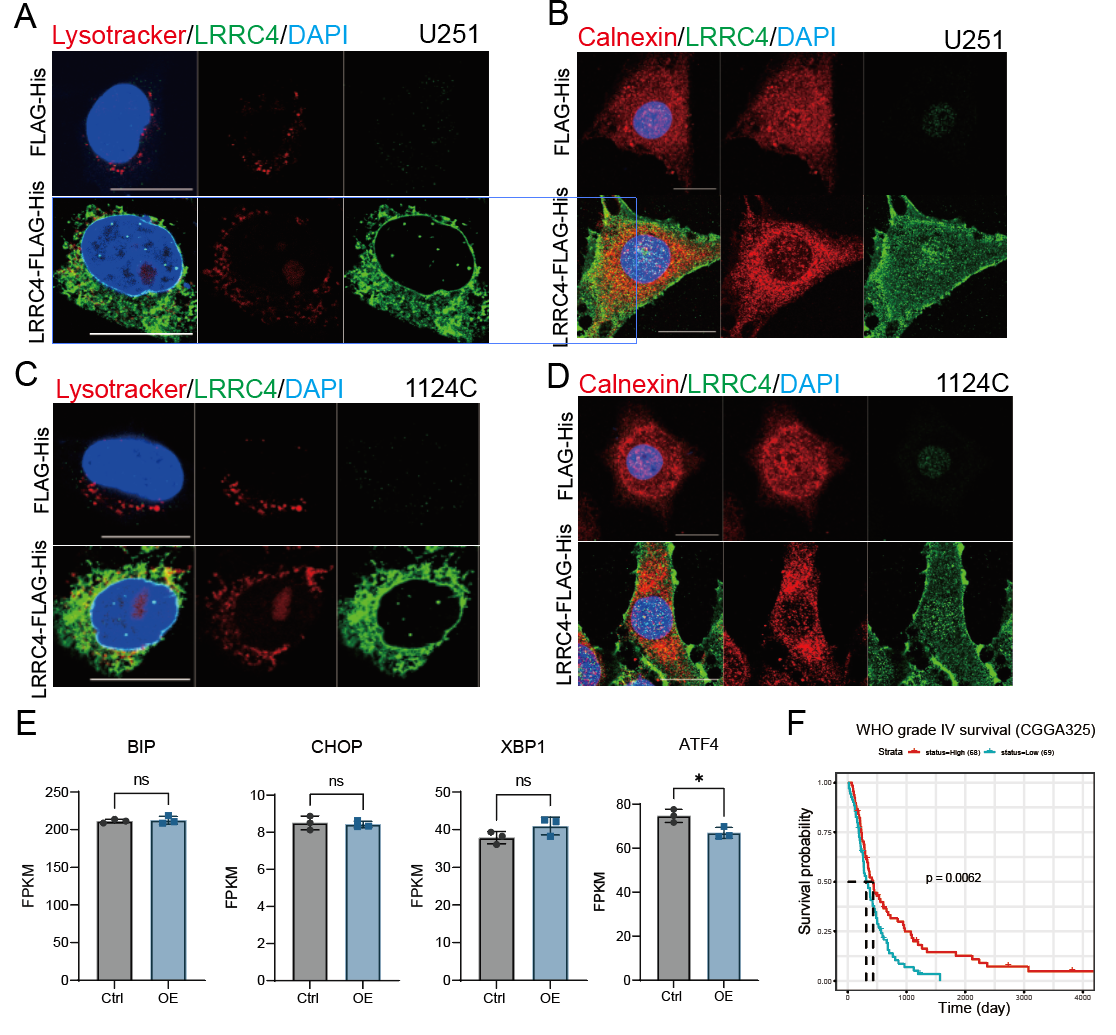


**Figure S1 LRRC4 overexpression maintains the homeostasis of the endoplasmic reticulum (ER) and lysosomes. A-D** Representative immunofluorescence images in U251 and 1124C cells. Exogenous LRRC4 (green) does not significantly co-localize with the ER marker Calnexin (red) or the lysosomal marker Lysotracker (red), indicating that LRRC4 is not a resident protein of these organelles. **E** mRNA expression levels of canonical ER stress markers measured by RNA-seq. Overexpression (OE) of LRRC4 fails to induce the expression of BIP, CHOP, or XBP1, while significantly reducing the expression of ATF4. This demonstrates that LRRC4 does not trigger the unfolded protein response (UPR) or ER stress. **F** Kaplan-Meier survival analysis of the CGGA325 cohort for WHO grade IV glioma patients shows that high LRRC4 expression is significantly associated with improved overall survival (p = 0.0062).


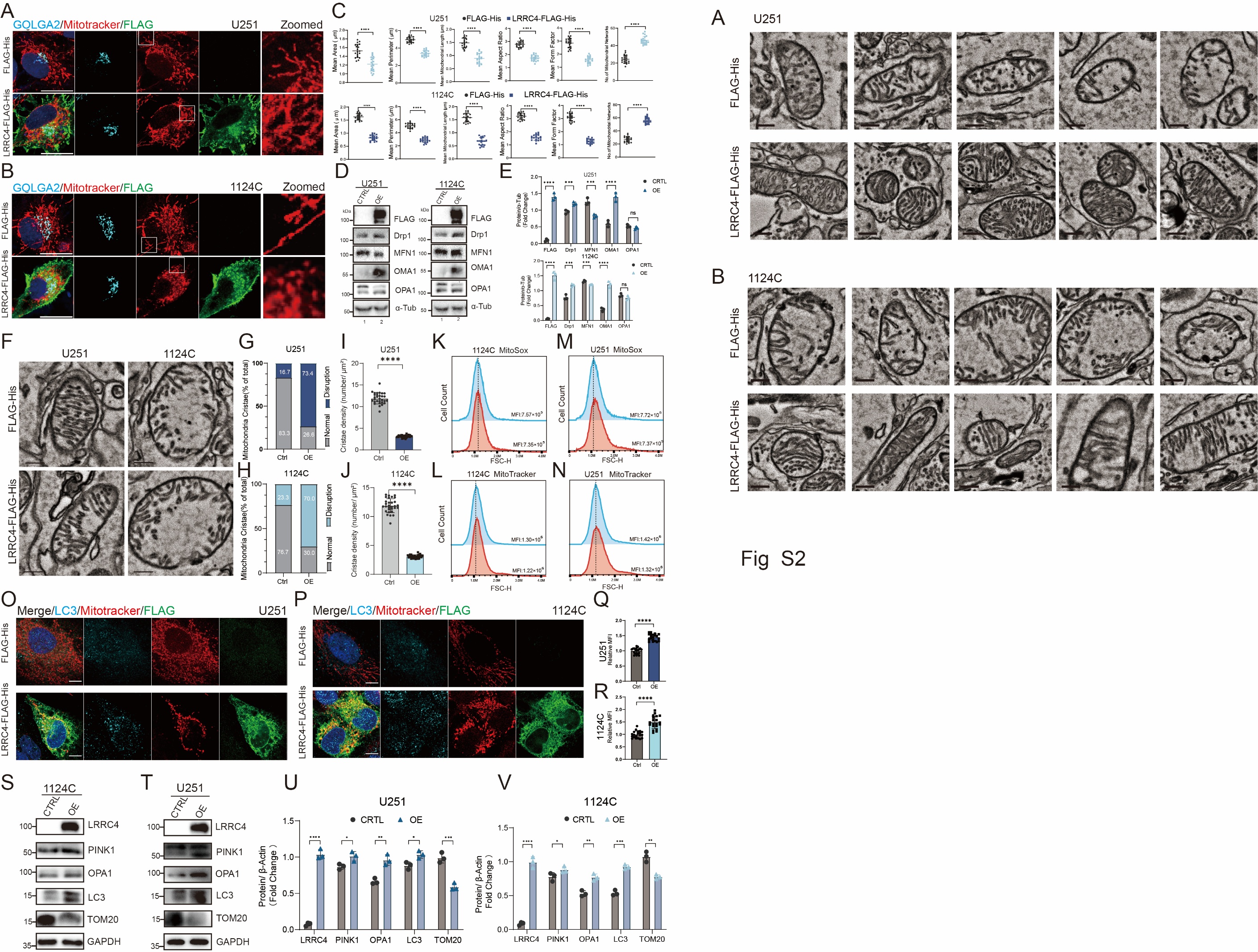


**Figure S2 TEM images for mitochondrial morphology evaluation.** **A-B** Representative high-magnification micrographs showing mitochondrial ultrastructure in U251 and 1124C cells expressing FLAG-His (Control) or LRRC4-FLAG-His (OE). These images served as the basis for quantifying cristae disruption and fragmentation. Scale bars = 200 nm; Magnification = ×20,000.


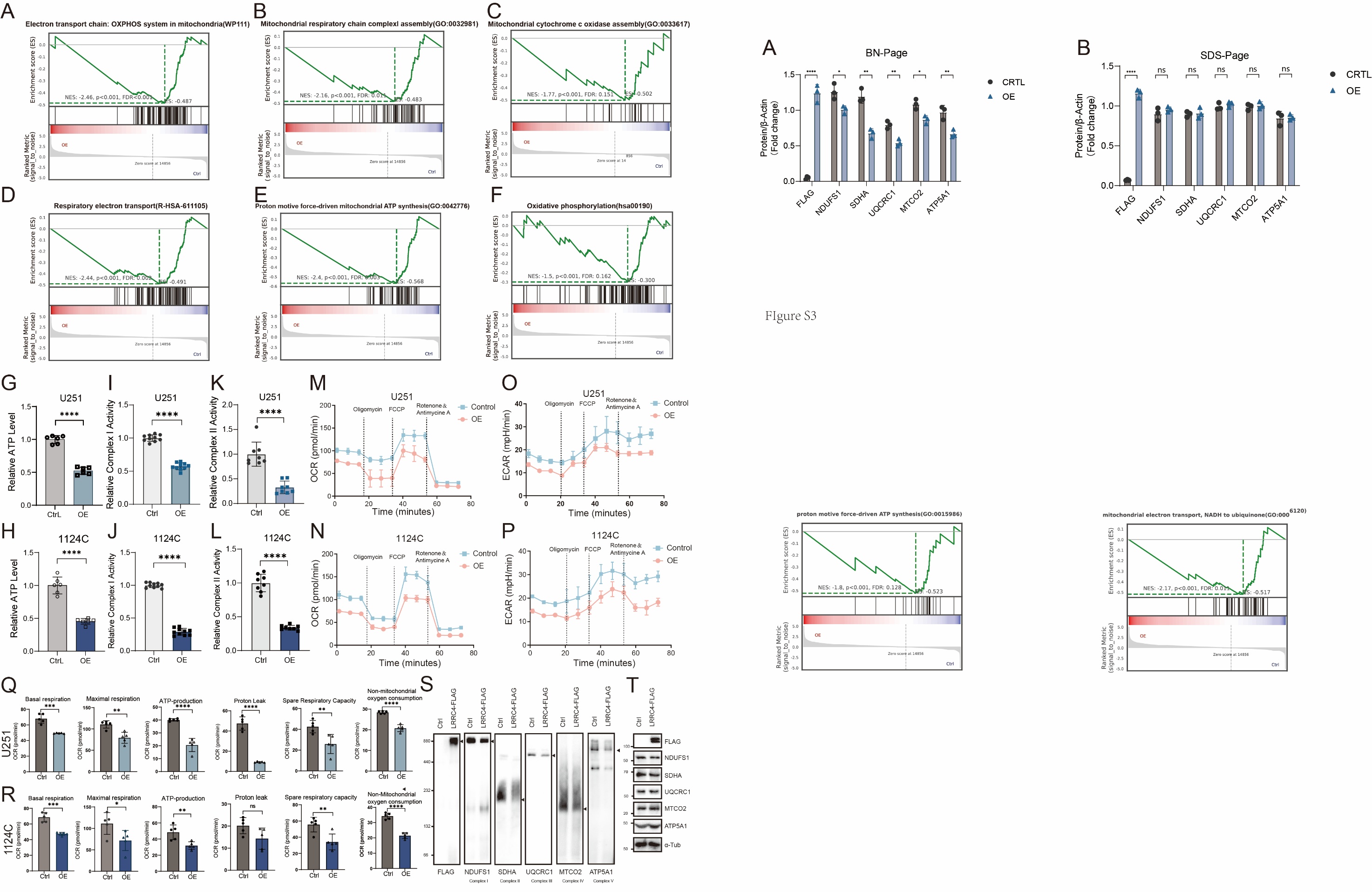


**Figure S3 Quantitative analysis of mitochondrial respiratory chain complex assembly and subunit expression**. **A** Quantitative analysis of the Blue Native PAGE (BN-PAGE) results from Figure 3S, demonstrating the relative abundance of assembled mitochondrial respiratory chain complexes (indicated by NDUFS1, SDHA, UQCRC1, MTCO2, and ATP5A1) in control (CRTL) and LRRC4-overexpressing (OE) cells. **B** Quantitative analysis of the SDS-PAGE results from Figure 3T, showing the relative total protein expression levels of the corresponding individual respiratory chain subunits in CRTL and OE cells. Data are presented as mean ± SD. * p < 0.05, p < 0.01, **** p < 0.0001, ns: not significant.


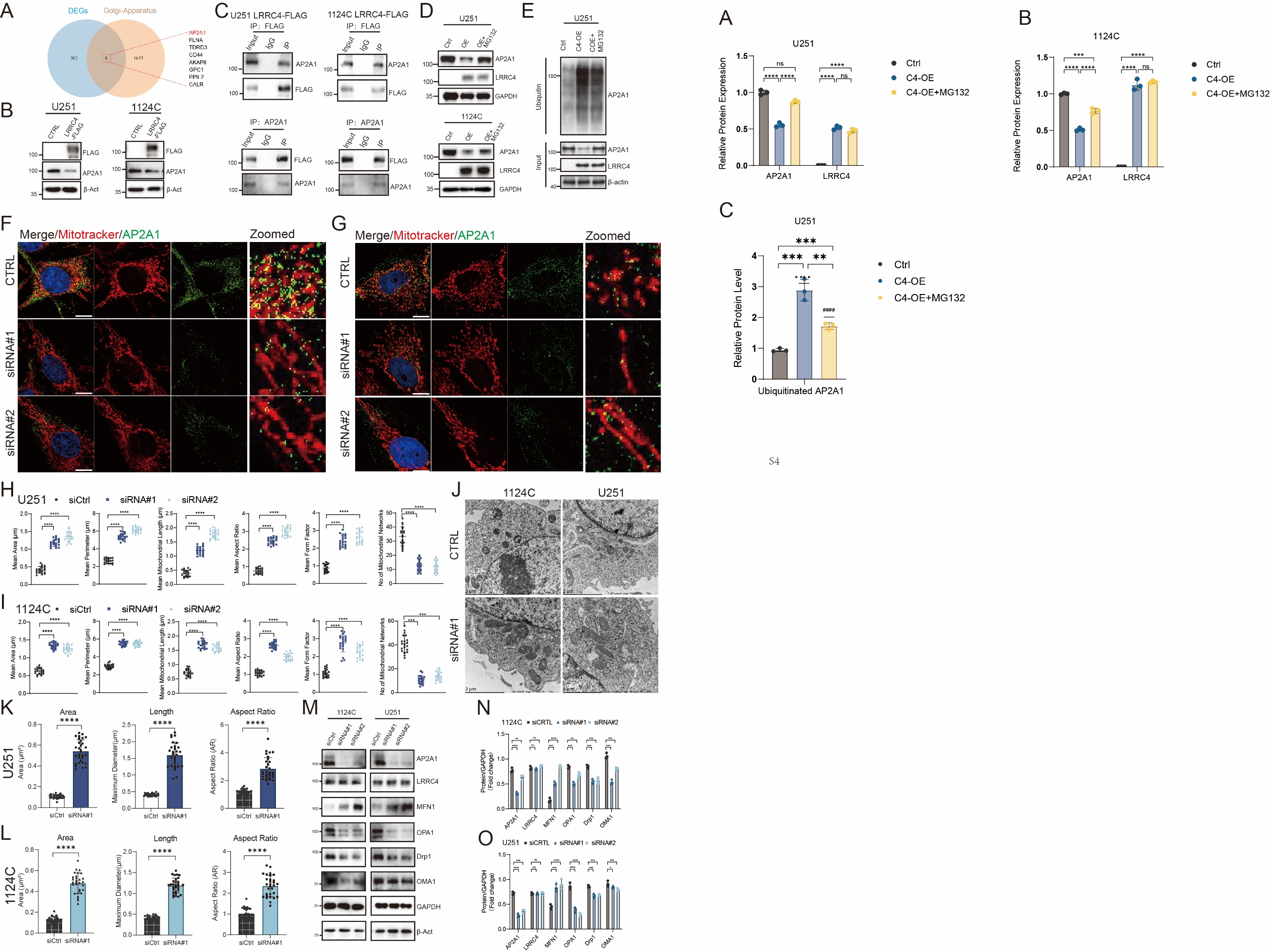


**Figure S4 Statistical quantification of AP2A1 degradation and ubiquitination.** **A-B** Quantification of Figure 5D showing that LRRC4-induced reduction of AP2A1 is effectively rescued by MG132 treatment in U251 (A) and 1124C (B) cells. **C** Quantification of Figure 5E confirming that LRRC4 overexpression significantly enhances the polyubiquitination of AP2A1 in U251 cells. Data are presented as mean ± SD; p < 0.01, *** p < 0.001, **** p < 0.0001, ns: not significant.


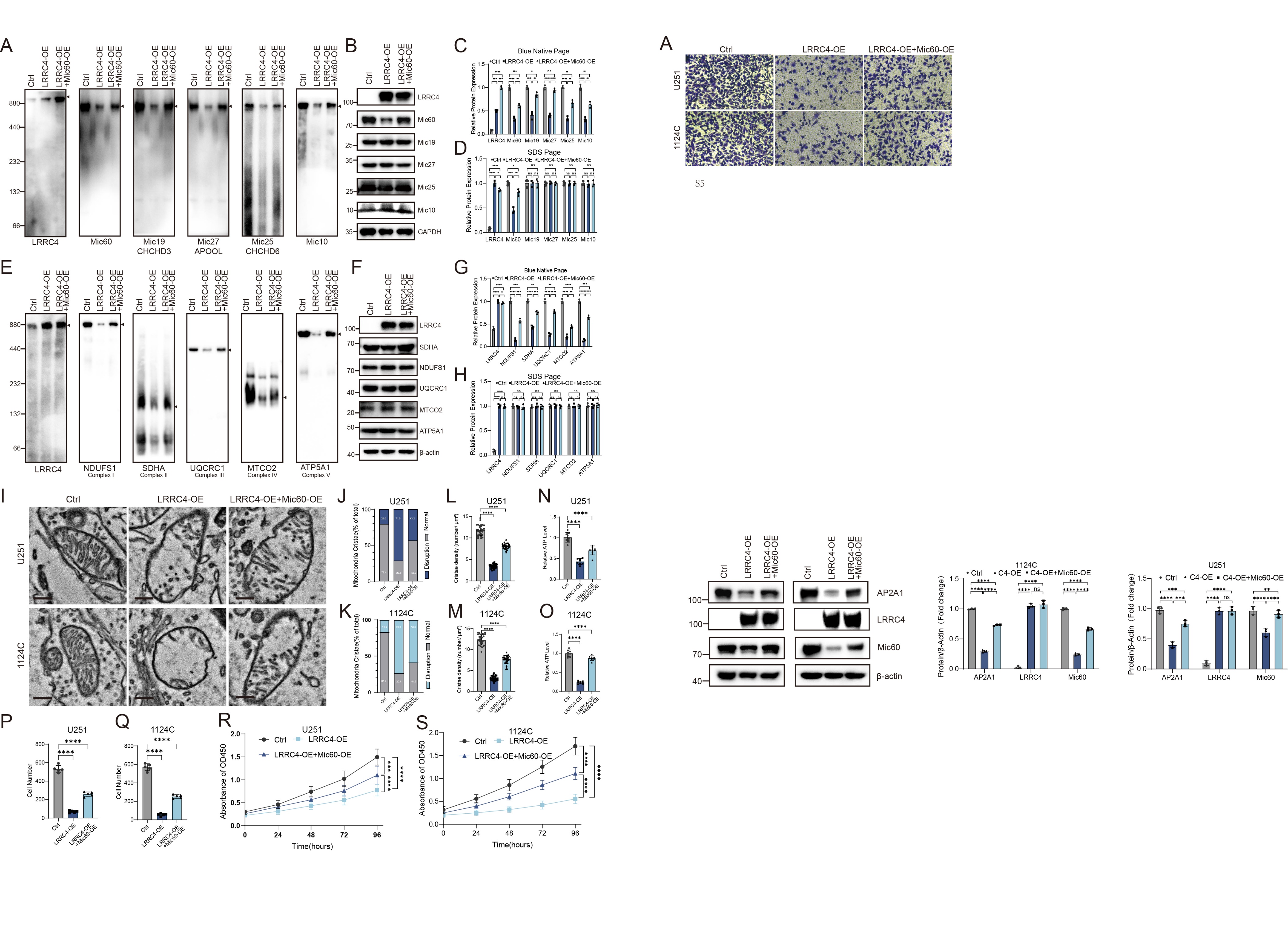


**Figure S5 Representative Transwell invasion images of U251 and 1124C cells under Ctrl, LRRC4-OE, and LRRC4-OE + Mic60-OE conditions.** **A** LRRC4 overexpression markedly reduced GBM cell invasion, whereas Mic60 re-expression partially restored the invasive capacity suppressed by LRRC4. Cells were stained with crystal violet. This supplement corresponds to the quantitative analyses shown in Fig. 7R–S.


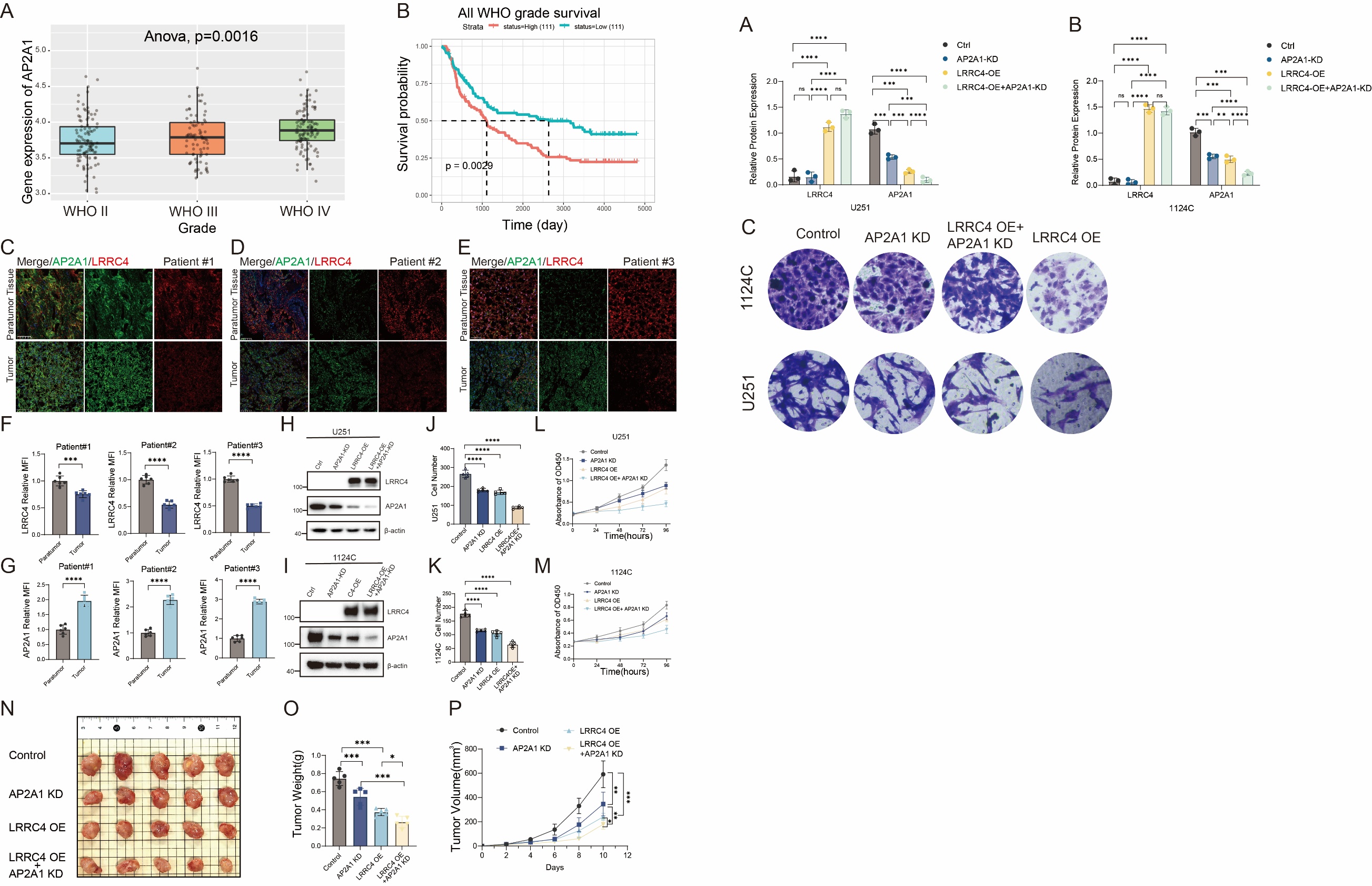


**Figure S6 LRRC4 and AP2A1 regulate GBM invasion and protein levels. A-B** Western blot quantification of LRRC4 and AP2A1 in U251 (B) and 1124C (C) cells corresponding to Fig. 8H–I. Data are mean ± SD from three independent experiments. Statistical significance: *p < 0.05, **p < 0.01, ***p < 0.001, ****p < 0.0001. **C** Representative Transwell invasion images of 1124C and U251 cells under Control, AP2A1 KD, LRRC4 OE, and LRRC4 OE + AP2A1 KD conditions.
